# Job loss disrupts individuals’ mobility and their exploratory patterns

**DOI:** 10.1016/j.isci.2025.112892

**Published:** 2025-06-13

**Authors:** Simone Centellegher, Marco De Nadai, Marco Tonin, Bruno Lepri, Lorenzo Lucchini

**Affiliations:** 1 Fondazione Bruno Kessler (FBK), Trento, Italy; 2 Department of Sociology and Social Research, University of Trento, Trento, Italy; 3 Centre for Social Dynamics and Public Policy, Bocconi University, 20100 Milan, Italy; 4 Institute for Data Science and Analytics, Bocconi University, 20100 Milan, Italy

**Keywords:** Social sciences, Human geography

## Abstract

In recent years, human mobility research has discovered universal patterns capable of describing how people move. These regularities have been shown to partly depend on individual and environmental characteristics (e.g., gender, rural/urban, and country). In this work, we show that life-course events, such as job loss, can disrupt individual mobility patterns. Adversely affecting individuals’ well-being and potentially increasing the risk of social and economic inequalities, we show that job loss drives a significant change in the exploratory behavior of individuals with changes that intensify over time since the job loss. Our findings shed light on the dynamics of employment-related behavior at scale, providing a deeper understanding of key components in human mobility regularities. These drivers can facilitate targeted social interventions to support the most vulnerable populations.

## Highlights

• Introduce a real-time method to infer individual unemployment using GPS trajectories
• Analyze the mobility of ∼1 M US individuals before and after job loss during COVID-19
• Reveal a sustained contraction in exploration that deepens with time since job loss
• Show demographic factors amplify mobility impacts, guiding targeted interventions

## Graphical abstract

**Figure:**
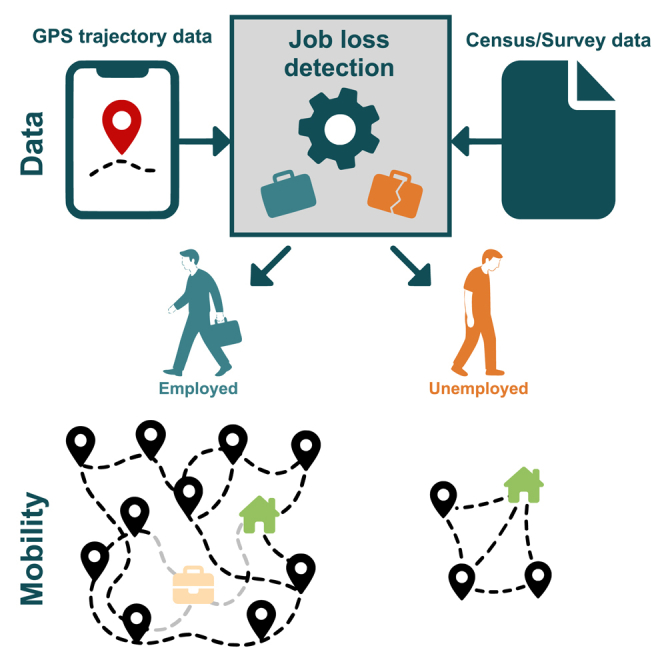

## Introduction

Economic and human behavioral statistics are crucial for effective decision-making. Large-scale population surveys have been invaluable in observing economic shocks and their implications. For example, unemployment data serve as a vital indicator of an economy’s health and performance^1^: When workers become unemployed, it affects their well-being and that of their families, it diminishes their purchasing power, and impacts the overall economy. However, conventional methods to track unemployment and its implications have been challenged by survey participation rates decline^2^^,^^3^ especially in developing countries.^4^^,^^5^

Recently, a transformative shift has emerged through the utilization of large-scale behavioral data collected from technologies like mobile phones, GPS trackers, social media platforms, and credit cards. This shift have been instrumental in advancing research in human mobility,^6^^,^^7^^,^^8^^,^^9^^,^^10^ financial well-being and purchase behavior,^11^^,^^12^^,^^13^^,^^14^^,^^15^^,^^16^ segregation and economic inequalities,^17^^,^^18^^,^^19^^,^^20^^,^^21^ crime,^22^^,^^23^^,^^24^ and public health.^24^^,^^25^^,^^26^^,^^27^^,^^28^

Few studies in human mobility research have managed to estimate job loss at a fine-grained level^29^^,^^30^^,^^31^ with even fewer studies focused on the behavioral specificities of unemployed individuals.^31^^,^^32^^,^^33^ However, the impact of job loss on individual mobility behavior at scale still remains a largely unexplored area of study.

In this context, the contribution of our work is 2-fold. First, we introduce a real-time methodology for inferring unemployment status based on individual GPS trajectories. Second, we provide evidence of significant changes in mobility behavior regularities following a job loss, particularly affecting vulnerable groups already at an increased risk of segregation.^20^

We leverage a dataset of privacy-enhanced longitudinal GPS mobility traces of nearly 1 million anonymous opted-in individuals from January 3, 2020, to September 1, 2020, across several US states. In order to preserve privacy, the data provider obfuscates devices’ home locations to the Census Block Group level and removes visits to sensitive points of interest from the dataset. The states are selected based on their diverse workforce composition profiles, enabling us to estimate unemployment at scale and analyze multiple facets of individuals’ mobility behavior following job loss. To ensure the representativeness of the GPS data and address potential sample biases,^21^ we employ a reweighting technique. This process generates a resampled cohort that reflects the demographic characteristics and the employed workforce across industrial sectors in all the states under study. We evaluate our methodology in the context of the COVID-19 pandemic, discussing its versatility for more general systemic shocks.

Our analysis sheds light on the impacts of job loss, providing a comprehensive, multidimensional view of individuals’ mobility patterns. This includes their geographic displacement, time allocation, and set of visited locations. We also show, through a temporal-independent analysis of employed versus unemployed behavioral patterns, that there is an increasing disparity in mobility behavior between employed and unemployed individuals since the time of job loss. In this perspective, we also illustrate how demographic factors such as sex, age, income, race, and education level can intensify the impact of job loss on individual mobility pattern contraction.

Overall, our results provide evidence for the long-term effects of unemployment on individuals’ daily lives. Job loss, as a major event in an individual’s life, not only perpetrates but also exacerbates existing socio-demographic disparities in mobility behaviors. While prior literature on human mobility has identified universal characteristics in mobility patterns,^34^^,^^35^^,^^36^^,^^37^ our findings highlight that individual life-course events, such as job loss, can affect these regularities at the individual level. Such events have the potential to influence people’s habits, as well as their social and psychological well-being.

## Results

### Inferring individual employment status

**Figure 1:**
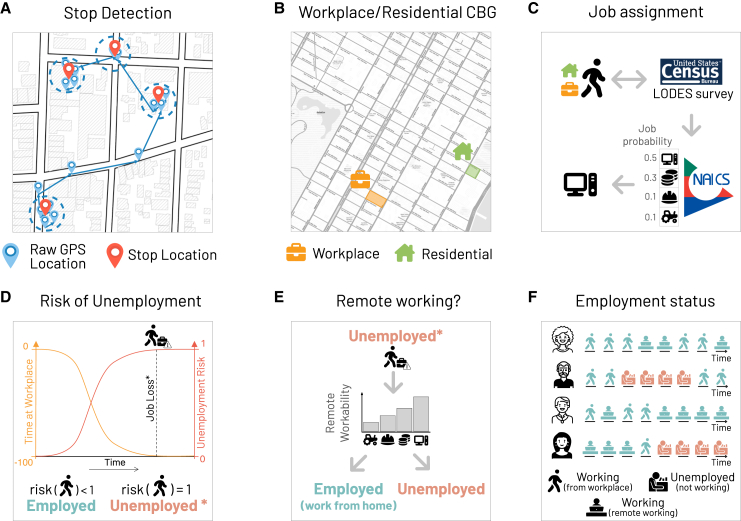
Employment status detection algorithm Overview of the developed procedure to detect unemployment. (A) Stop locations detection (individual stopped in a 65-meter radius and stayed for at least 5 min); (B) Workplace and Residential Census Block Groups (CBGs) detection; (C) Job Assignment, a NAICS sector is assigned in probability given the individual’s Workplace and Residential CBGs; (D) the Risk of Unemployment is computed for each individual based on their workplace visits; (E) Remote working correction based on the teleworkability of the individual job (NAICS sector); (F) For each individual we have their full employment status over time. Icons: Fontawesome, Flaticon, Maps: Stamen Maps.

To determine an individual’s employment status, we have devised a procedure that integrates both location and survey data (see (Figure 1)). We use a large and longitudinal dataset of privacy-enhanced GPS location data collected across seven US states from January to September 2020 to enrich census survey data provided by the United States Bureau. Particularly, we use the Longitudinal Employer-Household Dynamics (LEHD) Origin-Destination Employment Statistics (LODES),^38^ which provides privacy-preserved statistics about the US workforce divided by industrial sectors classified with the North American Industry Classification System (NAICS)^39^ and provides data about how many individuals are employed in a specific NAICS sector, based on their census block groups (CBGs)^40^ of residence and workplace. We refer to the STAR Methods section for further details.

For each individual, we first identify stop locations, defined as sequences of GPS coordinates within a 65-meter radius where a user stayed for a minimum of 5 min ((Figure 1A)). Then, after detecting the individual’s residential and workplace locations ((Figure 1B)), we enrich these locations with the LODES data information.

For those individuals with a detected work location, we label them as employed during the period in which their workplace location is identified. Each of these individuals is further assigned in probability a job, more specifically, an NAICS sector based on available survey data looking at their residential and workplace CBGs ((Figure 1C)).

As a following step, individuals are labeled as “at risk of unemployment” based on the reduction in visits to the workplace: if individuals never visit their workplace location, they are considered as potential candidates for unemployment ((Figure 1D)). The determination of employment status is sampled accounting for both the risk status and the NAICS-specific likelihood of working from home at any given time.

To account for whether an individual is working from home, we leverage information on the “teleworkability” of jobs, as presented in the study of Dingel and Neiman.^41^ For each industrial sector, the data provides the percentage of work that can be performed remotely ((Figure 1E)). Based on the individual’s job sector (NAICS), population-wide change in the time spent at work, and the weight of each individual in contributing to that change, we infer the unemployment status over time, thus determining whether the individual is working from home or is unemployed (see STAR Methods section).

### Cohort selection and algorithm evaluation

The privacy-enhanced location data provided by the location intelligence company Cuebiq intentionally excludes any direct information about users’ employment to safeguard privacy. This absence of direct job-related information presents challenges in establishing ground truth for individuals’ employment status. Consequently, we evaluate the accuracy of our methodology at an aggregate level by leveraging aggregated monthly statistics from Unemployment Insurance (UI) claims and Local Area Unemployment Statistics (LAUS) datasets (see Section S1 for dataset details). UI claims data offer near real-time information on the number of claimants, reported weekly, providing a timely basis for our algorithm evaluation. In contrast, LAUS data represent official unemployment figures derived through an estimation process that incorporates multiple sources, including UI claims, but are published and consolidated with a longer delay. Additionally, we incorporate state-level employment information from the Bureau of Labor Statistics (BLS) through the Quarterly Census of Employment and Wages (QCEW) program (see Section S1 for additional data details).

All our analyses are conducted on a cohort of mobile phone users residing in the United States. We employ individual reweighting to reconstruct a more representative cohort sample that mirrors both (1) population-wide representativeness, based on CBG population data and (2) representativeness of the employed workforce population in each state across various industrial NAICS sectors, drawing from state-level employment statistics (BLS statistics). This post-stratification procedure is crucial for addressing potential biases within the location data, improving data representativeness, and results reliability as recently discussed in the literature.^21^^,^^42^^,^^43^ While this approach cannot fully eliminate all possible sources of biases in mobile phone data, it enables us to compare employment status with Unemployment Insurance claims and LAUS unemployment and subsequently examine mobility patterns on a population-wide scale (see Section S2.1 for more details on the post-stratification procedure).

Our study focuses on seven US states: New York, Wyoming, Indiana, Idaho, Washington, North Dakota, and New Mexico. These states were selected to capture diverse workforce compositions spanning primary, secondary, and tertiary economic sectors, as well as geographic diversity across the United States (see Section S2.2 for more details).

To assess the reliability of our job detection methodology, we use both LAUS and UI claims data. The UI claims enable near real-time evaluation of the algorithm at the monthly level, segmented by NAICS sectors. At the state level across the seven studied states, we observe a Pearson correlation coefficient of 0.89 between the monthly rate of individuals detected as unemployed (reweighted to match the employed population) and the monthly UI claims rate. Using the official unemployment statistics from the LAUS data, we find a Pearson correlation of 0.72 at the state level and 0.53 at the county level. These results demonstrate that our algorithm is fairly reliable and can estimate unemployment at an aggregate level (see Section S4 for further details on the algorithm evaluation).

### Behavioral disparities between employed and unemployed individuals

The availability of inferred individual employment status data over time provides a unique opportunity to gain insights into the impact of job loss on human mobility. The wealth of information at our disposal allows us to characterize and quantify changes in behavior by comparing the daily mobility of individuals identified as employed or unemployed, offering a better understanding of shifts in mobility patterns following a job loss. In this study, we address two key questions: (1) How did individuals who experienced a job loss navigate through the pandemic period? And, more broadly, (2) what are the effects of job loss on an individual’s mobility behavior, and what happens when individuals face a prolonged period of unemployment?

To address these questions and ensure a fair comparison between a population of employed individuals and a population of unemployed individuals, we exclude all stop locations associated with an individual’s workplace from our analysis. Therefore, our analysis focuses on extra-work individuals’ mobility patterns, with a specific focus on those individuals who had been employed (even briefly) between January 3rd, 2020, and March 7th, 2020, namely before the WHO declaration of the COVID-19 pandemic (March 11th, 2020).

Considering systemic external factors in our analysis and given the significant stress placed on the labor market by the pandemic, we have the ideal conditions to study the consequences and gain a comprehensive understanding of the effects of job loss on individuals’ mobility behavior.

Note that, as previously explained, our analysis focuses on individuals who were employed, even briefly, before the pandemic. Therefore, the curves representing the mobility indicators for unemployed individuals in the baseline period may not be representative.

#### The disproportionate impact of the pandemic on unemployed individuals’ mobility

To provide a comprehensive analysis of changes in mobility, we measure within-individual variations by comparing activities to a baseline period preceding the pandemic (February 1st, 2020 – March 7th, 2020). Our focus revolves around three key well-known mobility metrics: (1) the radius of gyration ($r_{g}$),^6^ which measures the characteristic geographical displacement of individuals; (2) the time allocation entropy (*H*),^44^ which measures the distribution of time allocation in each visited location; and (3) the users’ locations’ capacity, denoted as *C*, which captures the number of a user’s familiar locations, alongside the number of locations added (*A*) to, and deleted (*D*) from, the set of familiar locations within a specific time interval^35^ (find the formal definition of the mobility metrics in the “STAR Methods” section). Collectively, these measures offer a multidimensional perspective on both the characteristic displacement and the complexity of individuals’ exploratory behavior.

**Figure 2:**
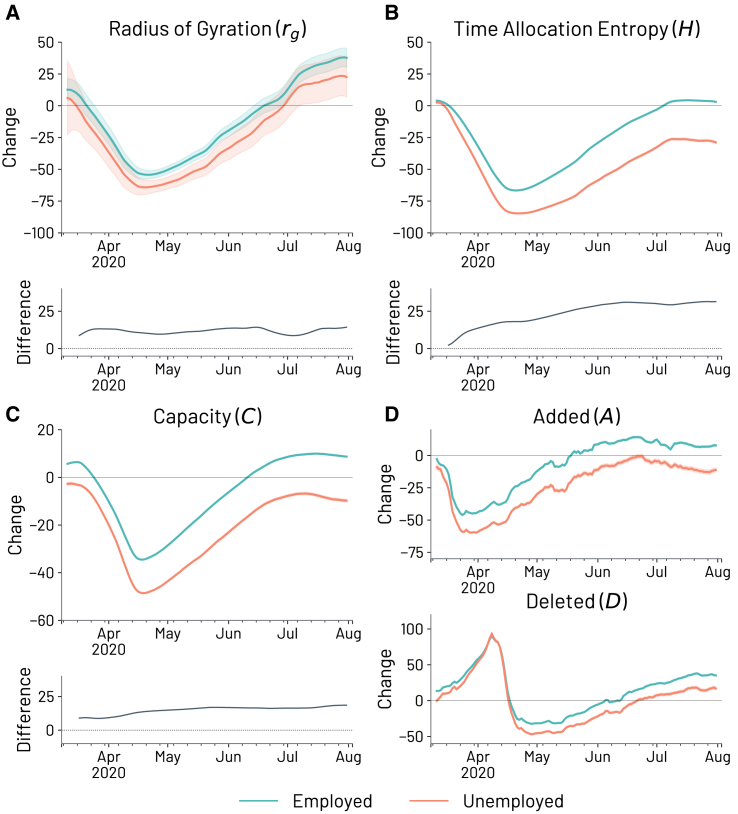
Impact of the pandemic on employed and unemployed mobility Percentage changes with respect to the baseline period (February 1st – March 7th) in extra-work individuals’ mobility patterns for employed and unemployed groups and their difference over time, as measured by different mobility metrics. (A) Radius of gyration ($r_{g}$) and the corresponding difference between the groups of employed and unemployed over time; (B) Time allocation entropy (*H*), which measures the distribution of time allocation in each visited location; (C) Capacity *C* which represents the number of a user’s familiar locations; and (D) the number of added *A* and deleted *D* locations between consecutive windows. Each mobility metric is computed over a window of 28 days with a 1-day shift. Shaded areas show the 2 standard error regions.

In (Figure 2), we present the results over time for each of these metrics and the relative difference over time between the group of employed and unemployed individuals. We consistently compare the mobility patterns of inferred unemployed individuals with those of employed individuals under similar pandemic-related conditions and restrictions. This approach helps isolate the specific effects of unemployment from broader external factors, such as lockdown measures. All the mobility metrics are computed for a window of 28 days with a 1-day shift. Note that, as previously explained, our analysis focuses on individuals who were employed, even briefly, before the pandemic. The results shown in Figure 2 reveal a substantial impact of the pandemic on individual mobility patterns, particularly among unemployed individuals. Notably, the group of unemployed individuals exhibits lower overall activity levels across all the mobility metrics under study. Moreover, we observe that as the pandemic progresses, the mobility gaps between the employed and unemployed groups widen. While the reduction in mobility for the unemployed is limited when examining the individuals’ characteristic displacement measured by the radius of gyration, with employed individuals reaching a low point of −54% and unemployed individuals reaching a low point of −64%, the same is not true when looking at regularity and exploration patterns. The drop in activity is particularly pronounced for unemployed individuals when examining the time allocation entropy, with low points of −70% and −87% for employed and unemployed individuals, respectively, and capacity, with low points of −37% and −51% for employed and unemployed individuals, respectively.

From this analysis, it becomes evident that the routine and exploratory behaviors of unemployed individuals, as measured by the time allocation entropy and by the capacity (together with its location turnover of added *A* and deleted *D* locations), were more affected than those of employed individuals. Moreover, over time, there is an evident increasing trend in the difference between the behaviors of the two groups. The difference between the two groups at the end of the period under study is 24% for the radius of gyration, 36% for the time allocation entropy, and 21% for the capacity. Interestingly, following the gradual reduction of COVID-19 restrictions, there appears to be a clear (partial) recovery for all the different facets of mobility behavior we analyzed.

#### Prolonged unemployment and the deterioration of mobility behavior

In the previous section, we provided insights into the collective mobility dynamics of employed and unemployed individuals, uncovering a disproportionate mobility response during the pandemic period between the two groups. To extend the validity of our findings beyond the pandemic conditions and ensure their generalizability to other possible systemic shocks, we further investigate into the growing divergence over time between the mobility behaviors of employed and unemployed individuals. Through the following analysis, we aim to understand the effects of job loss on individual-level mobility behavior, assessing whether a prolonged period of unemployment leaves a lasting impact.

Hence, we present a robust and general framework for detecting and tracking unemployment potentially adaptable to different systemic shocks. In particular, we propose a time-independent analysis of employed/unemployed behavioral patterns, which tries to understand whether the duration of unemployment contributes to the growing disparity between the two groups’ mobility behavior.

Due to the period during which the data were collected, we first need to consider the non-negligible impact of Non-Pharmaceutical Interventions, and more in general of the pandemic, on the general population mobility during 2020. To mitigate the effect of the COVID-19 pandemic on the results, we standardize each individual’s mobility indicator by calculating the *Z* score using the average and standard deviation of the employed group’s indicators on a specific day *t*. Then, to better understand the effects of a job loss on an individual, we align the mobility indicators of all individuals by shifting time so that t=0 represents the time when an individual lost their job. This approach enables consistent comparisons of individuals’ mobility behavior at different times with respect to the date on which they lost their jobs.

**Figure 3:**
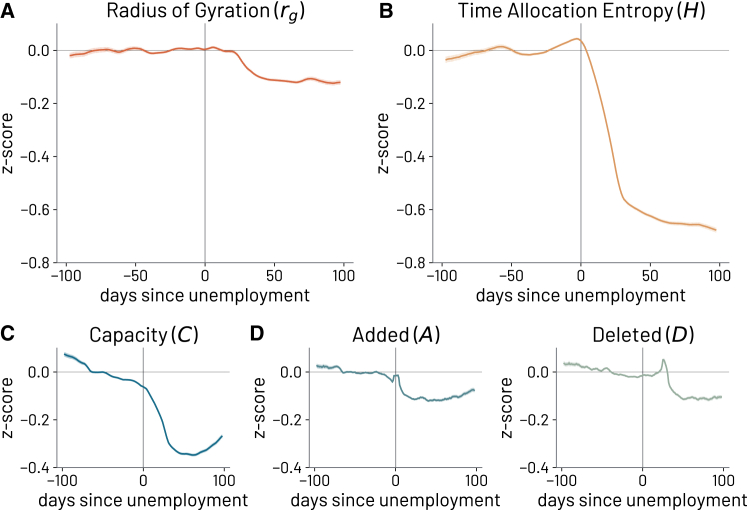
Individual-level mobility behavior after job loss using employed population as reference group The results show the lasting impact of prolonged periods of unemployment on individual-level mobility behavior. Each individual’s mobility indicator, which includes (A) the radius of gyration, (B) the time allocation entropy, (C) the capacity *C*, and (D) the added *A* and deleted *D* locations over time, is standardized by calculating the *Z* score using the average and standard deviation of the employed group’s indicators on a specific day *t*. Then, time is aligned such that at time t=0, individuals have lost their jobs. Shaded areas represent the 2-standard-deviation range.

As illustrated in (Figure 3), both the radius of gyration (at a smaller level) and time allocation entropy (at a larger level) were affected and gradually decreased over time, reaching almost −0.15 and −0.8 standard deviations, respectively, compared to when individuals were employed. Although the radius of gyration seemed to be less affected, the relative time allocation entropy of individuals who lost their jobs decreased sharply and constantly the longer they were unemployed. This large reduction in time allocation entropy may be related to the tendency of unemployed individuals to spend a significant fraction of time at home.^32^^,^^45^

A similar dynamic is observed in the capacity *C* of individuals, which displays a sharp decrease of more than −0.3 standard deviations, followed by a slow recovery after approximately 60 days. The added locations (*A*) to the set of familiar places exhibited similar behavior as the capacity, with a noticeable decrease after an individual loses their job. On the other hand, the deletion of familiar locations (*D*) increases abruptly when individuals lose their jobs, followed by a sharp decrease. Both the added and deleted locations then remain significantly low, indicating an overall lower turnover in the set of an individual’s familiar locations. Despite a modest recovery after approximately two months, the results highlight the clear and persistent impact of unemployment on limiting individuals’ abilities to explore new opportunities in physical space. The drop in capacity (*C*), together with the decrease in the number of added (*A*) and deleted (*D*) locations, highlight a reduced location turnover and a sustained contraction into the individuals’ set of familiar locations. For an individual, this scenario may indicate a potential decrease in exposure to opportunities and an increased risk of isolation after experiencing a job loss.

### Socio-demographic factors in job loss behavioral changes

To get a better understanding of the implications of prolonged unemployment, we evaluate and quantify socio-demographic differences in mobility patterns among individuals enduring a prolonged period of unemployment. Leveraging socio-demographic information from the Longitudinal Employer-Household Dynamics (LODES) dataset,^38^ including *Sex*, *Age*, *Income*, *Race*, and *Education*, we analyze demographic differences in mobility behaviors.

Building on the results presented in (Figure 3), we disaggregate the mobility behavior of unemployed individuals (t>0) based on the individual’s socio-demographic group (see Section S5 for more details), revealing significant disparities in the mobility behavior of unemployed individuals when compared with the mobility behavior of employed individuals (see Table S3).

**Figure 4:**
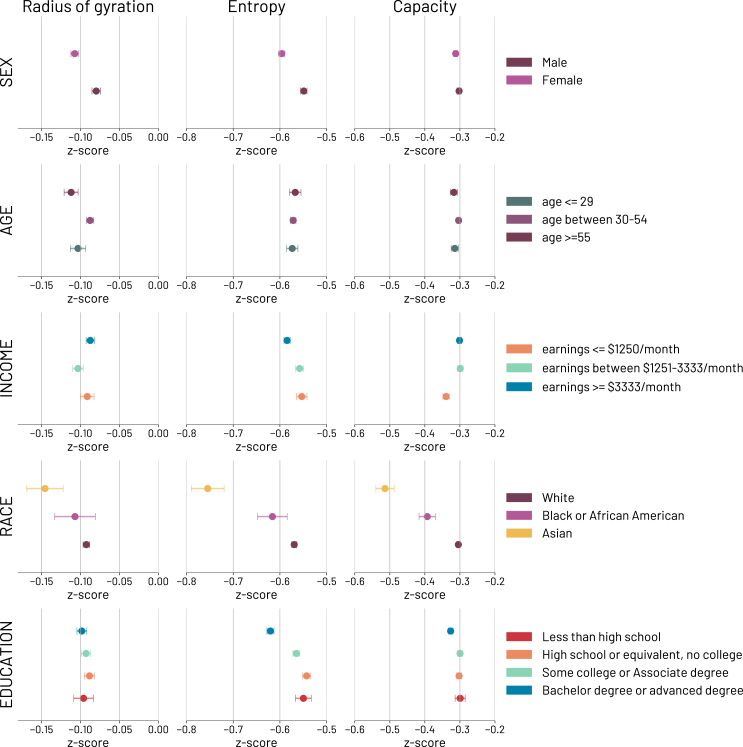
Demographic variations in mobility among individuals enduring a prolonged period of unemployment Differences in mobility behavior of unemployed individuals in the latter stages of unemployment (between 30 and 100 days) for Sex, Age, Income, Race, and Education demographics compared to the mobility indicators of the reference group of employed individuals. For each metric and demographic group, we provide the mean and standard error of each group in the 30–100 days period after the job loss.

In (Figure 4), we compare each mobility indicator of individuals who fall in a particular socio-demographic category against the population of employed individuals. The results show significant differences between male and female individuals across all three mobility indicators, namely radius of gyration ($r_{g}$), time allocation entropy (*H*), and capacity (*C*). Unemployed women generally exhibit lower values of mobility exploration ($r_{g}$, and *C*) and diversity (*H* and *C*). Regarding individuals’ *Age*, differences in mobility behavior are relatively smaller, with older individuals (age≥55) showing a more pronounced reduction in their characteristic geographical displacement ($r_{g}$) compared to other groups. *Income* disparities reveal smaller differences in the radius of gyration ($r_{g}$), whereas richer individuals (earnings≥$3333/month) exhibit lower values in their time allocation entropy (*H*). Capacity (*C*), in contrast, is lower for those individuals reporting lower income values (earnings≤$1250/month). In terms of *Race*, *Asians* display lower values in all three mobility metrics, followed by *Black or African American* individuals and then *White* individuals. Specific ethnic groups (e.g., *American Indian or Alaska Native*, *Native Hawaiian or Other Pacific Islander*, and *Two or More Race Groups*) have been excluded from the analysis due to small sample sizes. *Educational* levels show fewer differences in mobility behavior between groups, with no significant differences in radius of gyration ($r_{g}$). Lower values of time allocation entropy (*H*) and capacity (*C*) are observed in individuals with *Bachelor degree or advanced degrees*.

We test the significance of the differences between demographic groups using Welch’s t-test^46^ (see Table S4), considering the behavioral information from 30 days after job loss up to 100 days (to remove the initial transitioning phase).

To validate our understanding of job loss as an important life-course event that can shape individual mobility patterns, we conducted a comparative analysis between unemployed individuals and their employed counterparts from the same socio-demographic group. This comparison demonstrates that the reduced mobility behavior occurring after a job loss is consistently present, although with different intensity, in all the studied population strata (see Table S5 for the statistics).

Taken together, these results substantiate the interpretation of socio-demographic characteristics as a factor to be taken into account when aiming to mitigate the effects of unemployment on individuals in mobility patterns. The differences observed in mobility indicators after a job loss are attributable to both the transition to joblessness and the inherent mobility tendencies within socio-demographic groups.^47^^,^^48^^,^^49^^,^^50^

## Discussion

The availability of massive digital traces collected through mobile phones has become an important proxy for studying individual behavior at population scales. The size and granularity of these datasets have revealed crucial insights into the regularities of human mobility and have exposed universal properties of human mobility patterns.^9^^,^^35^^,^^36^^,^^37^^,^^44^^,^^51^^,^^52^ Interestingly, within the numerous mobility models for human mobility, the notion of “opportunities” consistently emerges as a key driver of individual movement patterns.^6^^,^^8^^,^^51^^,^^52^^,^^53^^,^^54^^,^^55^ This notion suggests that individuals navigate physical space in pursuit of various kinds of opportunities spanning social, educational, and economic domains.

In this perspective, understanding whether individuals transitioning to a state of unemployment can still access and benefit from the opportunities that their social and physical environments offer is of great social importance.^56^^,^^57^^,^^58^

To proxy social exposure and access to opportunities, we leverage an individual-level longitudinal dataset of fine-grained mobility behavior alongside secondary demographic data.^39^^,^^40^^,^^41^^,^^59^ We employ reweighting and rescaling techniques to address potential sample biases in the GPS data and to mitigate the effects of COVID-19 restrictions on mobility behavior analysis.

The empirical evidence we present highlights that individuals facing unemployment significantly decrease their mobility, suggesting a reduction in their ability to explore and exploit available opportunities. This effect worsens over time, leading to a differentiation of the population into employed and unemployed subgroups with persistent behavioral differences. In particular, the impact of job loss manifests differently across various socio-demographic groups, highlighting how some of these already vulnerable communities may be disproportionately affected.^47^^,^^48^^,^^49^^,^^50^^,^^58^

In this context, our work underscores the significant influence of personal circumstances or life events, such as job loss, on established patterns of human mobility. These life-course events can drive individuals to transition through different states of human mobility regularities, adding a layer of complexity to the notion that mobility patterns can depend on the structure of the surrounding physical space^9^^,^^33^^,^^60^ and the demographic attributes of individuals.^9^^,^^33^^,^^37^

Furthermore, for an individual, the reduction in exploration patterns is not only a reflection of the immediate impact of job loss but potentially also signals a broader issue, leading to a decreased exposure to opportunities and an increased risk of social isolation after experiencing job loss,^61^^,^^62^^,^^63^^,^^64^ reinforcing negative effects on an individual’s well-being.^65^^,^^66^^,^^67^^,^^68^

Importantly, the progressive reduction in individual mobility and the associated decline in social participation^61^^,^^62^^,^^63^^,^^64^ could also undermine the potential effectiveness of intervention programs targeting the early stages of unemployment. However, while our analysis focuses primarily on mobility and behavioral impacts of unemployment, important future directions include examining how these quantities can serve as early proxies of long-term socio-economic outcomes such as career trajectories, mental health, and social integration. By leveraging real-time data, our approach can facilitate targeted efforts during these initial phases, enabling more effective mitigation of the enduring and group-specific impacts of job loss.^57^^,^^58^

### Limitations of the study

In the field of human mobility and GPS analytics, it is recognized that mobile phone and GPS location data come with inherent limitations and biases. Although these datasets are rich and detailed from a temporal and spatial point of view, they often suffer from representativeness issues, such as the underrepresentation of younger and older age groups, or populations in low-income settings. Additionally, due to privacy reasons, such datasets typically lack associated demographic and socioeconomic metadata, including in our case individual-level employment information. As discussed in prior work,^21^^,^^43^ we address this challenge through reweighting techniques to get a better representation of the population in terms of demographic and employment characteristics.

Additionally, while our framework is flexible and adaptable to various contexts and data sources —emphasis that it is not a ready-made solution for any country in the world— it must be carefully validated and adjusted based on the specific characteristics of each region or country where it is applied. For example, the availability of supporting datasets, such as LODES data used in our study, is not accessible in every country and unemployment statistics are often reported with different levels of temporal granularity compared to the United States. Moreover, in the context of developing countries (and particularly for low-income countries),^42^ GPS-based methods may be less suitable due to lower smartphone penetration rates. In such settings, alternative data sources like Call Detail Records (CDRs) could provide more effective means for capturing employment-related mobility patterns.

## Resource availability

### Lead contact

Requests for further information and resources should be directed to and will be fulfilled by the lead contact, Lorenzo Lucchini (llucchini@fbk.eu).

### Materials availability

This study did not generate new materials.

### Data and code availability

• The data supporting the findings of this study are accessible through Cuebiq’s Data for Good initiative. For details on how to request access, including conditions and limitations, please visit: https://www.cuebiq.com/about/data-for-good/.
• Replication code is available on GitHub at https://github.com/scentellegher/ImpactJobLoss/.
• Any additional information required to reproduce the results of this paper is available from the lead contact upon request.

## Acknowledgments

The authors would like to thank Cuebiq that kindly provided us with the mobility dataset for this research through their Data for Good program. L.L. thanks G.K. for the insightful discussions and his support during the entire project development. L.L. has been supported by the ERC project “IMMUNE” (Grant agreement ID: 101003183). L.L. acknowledges the support from the “10.13039/501100012032Fondazione Romeo ed Enrica Invernizzi” for the research activities of the “Covid Crisis Lab” at 10.13039/501100006375Bocconi University. S.C. and B.L. have been supported by the PNRR ICSC National Research Centre for High Performance Computing, Big Data and Quantum Computing (CN00000013), under the NRRP MUR program funded by the 10.13039/100031478NextGenerationEU.

## Author contributions

L.L., S.C., and M.D.N. conceived the original idea and planned the experiments. S.C., L.L., and M.D.N. pre-processed the mobility data. S.C., L.L., and M.T. carried out the experiments and made the figures. L.L., S.C., and M.D.N. contributed to the interpretation of the results. L.L. and S.C. wrote the manuscript. S.C., M.D.N., M.T., B.L., and L.L. provided critical feedback, helped shape the manuscript, and substantively revised it.

## Declaration of interests

The authors declare no competing interests.

## STAR★Methods

### Key resources table

**Table:** 

REAGENT or RESOURCE	SOURCE	IDENTIFIER
**Software and algorithms**		

Replication Code	https://github.com/scentellegher/ImpactJobLoss	

### Experimental model and study participant details

This study is based on privacy-enhanced mobile phone GPS trajectories provided by Cuebiq within the “Data For Good” Program. In our work, we report results drawn from a final sample of around 476k unique devices.

### Method details

#### Stop locations

The GPS location data was provided by Cuebiq, a location intelligence company that provided through their Cuebiq Data for Good COVID-19 Collaborative program, a dataset of privacy-enhanced GPS locations from users who opted-in to share the data anonymously for research purposes through a CCPA (California Consumer Privacy Act) compliant framework (see Section S1 for more details). To further preserve privacy, the data provider obfuscates users’ precise home and work locations by transforming them to the centroid of the corresponding Census Block Group. We analyze a dataset that spans a period of 9 months, from January 2020 to September 2020, for seven US states, including New York, Wyoming, Indiana, Idaho, Washington, North Dakota, and New Mexico.

We filter out all users with less than one month of data before declaring a national emergency (March 13, 2020) and less than four months after it. We also require users to have 5 hours per day covered by at least one GPS location. The resulting dataset includes more than 1 million anonymous, opted-in individuals. For all users, we extract their stop events with an algorithm based on Hariharan and Toyama.^69^ We define a stop event as a temporal sequence of GPS coordinates in a radius $\Delta_{s}=65$ meters where a user stayed for at least $\Delta_{t}=5$ minutes. For each user, we then define their stop locations as the stop events that can be considered as part of the same place using the DBSCAN algorithm.^70^ With DBSCAN, we group points within a distance of $\varepsilon =\Delta_{s}-5$ meters to form a cluster with at least minPoints=1 stop event. For a more detailed explanation of the GPS data processing, please refer to Lucchini et al.^64^

#### Residential and workplace detection

We determined the most likely residential and workplace areas for each user by calculating these areas multiple times over a moving rolling window of 28 days. We then aggregate for each day *t*, for each user *u*, and stop $s_{u}\left(t\right)$, the amount of time spent in a window [t−14,t+14] distinguishing between:

(1) **Residential time:** The amount of time between 8 pm and 4 am spent by the user *u* at stop $s_{u}$. Unlike previous studies such as TimeGeo,^71^ we did not assume the entire weekend as residential time since the US Bureau of Labor Statistics recently estimated that around 34% of employed people work in the weekend.^72^
(2) **Workplace time:** On weekdays, the amount of time between 9 am and 5 pm spent by the user *u* at stop $s_{u}$. We chose these working hours because they represent the most common working time in the US.^73^ Additionally, we assumed that a potential workplace stay should last at least 30 minutes and occur five times a week. These assumptions were similar to those made in previous studies.^71^

We detect for each user *u* their **residential location** as the stop location $s_{u}\left(t\right)$ with the largest *Residential time* during the period that goes from January 3rd to March 7th (before the pandemic). To detect changes in the **workplace location** for a particular user *u*, we label a stop $s_{u}\left(t\right)$ as workplace location if this stop is not a residential location (to avoid tracking people already working from home) and it has the largest *Workplace time* in the observed 28 days window. To protect users’ privacy, the residential and workplace locations were blurred and associated with the corresponding Census Block Groups.

#### Job assignment

The location data does not have direct information about the users’ jobs. To the extracted residential and workplace locations, we associate a Geographic Identifier (GEOID), which is a numeric code that uniquely identifies an administrative geographic area in the US Census data. To be able to assign a job to each user, we match the residential and workplace GEOIDs to the GEOIDs of the Longitudinal Employer-Household Dynamics Origin-Destination Employment Statistics (LODES) datasets.^38^ Given the residential/workplace locations, these datasets provide statistics about the number of jobs in each sector as defined by the North American Industry Classification System (NAICS).^39^

The information of the LODES datasets is organized into i) Residence Area Characteristics (RAC); ii) Workplace Area Characteristics (WAC); and iii) Origin-Destination (OD). RAC and WAC datasets provide job statistics according to the residential and workplace census block groups, respectively. The OD dataset provides job statistics considering both residential and workplace census blocks (for further details on the LODES datasets, refer to Section S1). From these three datasets, we then compute the probabilities of working in a particular NAICS sector by normalizing the number of jobs in each NAICS sector by the total number of jobs.

Finally, for each individual and the combination of their residential and workplace GEOIDs, we assign an industrial sector in probability. The probability of working in a specific sector, *i*, is computed for each user *u* with a home location and a work location, as the joint probability of independently working in that specific sector, given that *u* resides in *r* and works in *w* (their home and work locations respectively):

$$P\left(i,u|r,w\right)=P_{RAC}\left(i,u|r,w\right)\cdot P_{WAC}\left(i,u|r,w\right)\cdot P_{OD}\left(i,u|r,w\right)$$

where *r* is the residential GEOID and *w* is the workplace GEOID as provided in the LODES data.

Bootstrapping is applied for a more robust NAICS assignment to individuals. Specifically, for each individual, we sample 10 times from the corresponding NAICS probability distribution and retain all the sampled NAICS as independent realisations of a GEOID-representative population.

#### Employment status inference

To infer the employment status of a bootstrapped user, we leverage information on the reduction in i) workplace visits and ii) time spent at work.

##### Who is at risk of unemployment?

We define an individual to be at risk of losing their job if they never visited their work location. Since we are interested in studying the impact of job loss at the individual level, we restrict our analysis to individuals who were employed before the pandemic declaration. Using the pre-pandemic period as a baseline period makes it possible to investigate the shock induced by the pandemic on the job market. Specifically, we retain only users who have been working at least 5 days during the baseline period (t=b), namely the period before the pandemic (January 3rd - March 7th, 2020). We then compute the reduction of workplace visits and identify as “at risk” those who at a specific time window *t* didn’t visit their workplace. To identify the population at risk of unemployment over time, we used a time window of 28 days with a daily shift. Thus, at each time *t*, we define an individual to be at risk of unemployment, $r_{u}\left(t\right)$, if they didn’t visit their work location in the entire time window:

$$\left\{ \begin{array}{cc} r_{u}\left(t\right)=1 & \text{if}\;v_{u}\left(t\right)=0\\ r_{u}\left(t\right)=0 & \text{otherwise} \end{array}\right.$$

with $v_{u}\left(t\right)$ representing the number of visits a user *u* made to their work location within the time window *t*.

##### Who is working remotely?

Under the assumption that individuals at risk of unemployment ($r_{u}\left(t\right)=1$) could be working remotely, we assign to each individual the likelihood of working remotely based both on their personal and other workers’ (in the same NAICS) working behaviour changes. Individual working behaviour change is measured in terms of the reduction in the time spent at work with respect to the baseline period:

$$rt_{u}\left(t\right)=1-\frac{w_{u}\left(t\right)}{w_{u}\left(b\right)}\text{.}$$

Here $w_{u}\left(t\right)$ is the time the user *u* spent at the workplace in the time window *t*, and $w_{u}\left(b\right)$ is the median of the time spent at work (within windows of the same size as *t*) during the baseline period (January 3rd - March 7th, 2020).

By additionally adjusting for how much the entire sector *s* is working remotely during a specific window compared to the estimated maximum amount of time that can be worked remotely,^41^ we can write the probability of being unemployed as:

$$P_{s}\left(t\right)=1-\min\left(\frac{{\tilde{R}}_{s}\left(t\right)}{{\tilde{r}}_{s}\left(t\right)},1\right)$$

where ${\tilde{r}}_{s}\left(t\right)$ represents the weighted fraction of remaining work time that could be performed remotely by those individuals who stopped visiting their work location, and ${\tilde{R}}_{s}\left(t\right)$ represent the fraction of work that could be performed remotely adjusted by potential changes in remote working behaviour among those individuals who didn’t interrupt visiting their workplace (for additional details see Section S3.1).

Intuitively, by estimating at the sector level the reduction in the time spent at work by users who are still visiting their work location ($r_{u}\left(t\right)<1$), we measure how much “remote work” is already performed by individuals who are still visiting their workplace. The remaining part of the remote-workable time (if any), ${\tilde{R}}_{s}\left(t\right)$, is used to uniformly distribute the probability of being unemployed among individuals who stopped visiting their workplace (for further details see Section S3.1).

### Quantification and statistical analysis

#### Mobility metrics

To track the changes in mobility of employed and unemployed individuals, we measure within-individual variations by comparing mobility behaviours to a baseline period preceding the pandemic (February 1st, 2020 - March 7th, 2020). The mobility metrics we used in our analysis offer a comprehensive picture of mobility behaviour including individuals’ characteristic displacement and the complexity of individuals’ exploratory behaviour. To track the changes over time, we computed these metrics over a moving window of 28 days with a 1-day shift.

##### Time allocation entropy

We introduce the time allocation entropy, which measures the distribution of time allocation in each visited location by an individual, as a measure of exploratory behaviour: $H\left(i\right)=-\frac{\sum_{j=1}^{k}p_{ij}\;\log\left(p_{ij}\right)}{\log\;k}\text{.}$ Here, *k* is the total number of unique visited locations of an individual *i*, $p_{ij}=\frac{V_{ij}}{\sum_{j=1}^{k}V_{ij}}$ and $V_{ij}$ is the total time individual *i* spends in location *j* (weighted by time spent).

##### Radius of gyration

To measure the characteristic geographical displacement of individuals, we use the well-known radius of gyration^6^^,^^7^ defined as: $r_{g}=\sqrt{\frac{1}{N}\sum_{l\in L}n_{l}{\left|r_{l}-r_{\text{cm}}\right|}^{2}}$, where *N* is the total time spent by a particular individual to all their visited locations; $n_{l}$ is the time spent to location *l*; *L* is the set of stop locations within a time window; $r_{l}$ is a two-dimensional vector representing the location’s GPS position recorded as latitude and longitude; and $r_{\text{cm}}$ is the centre of mass of the trajectories, defined as $r_{\text{cm}}=\frac{1}{N}\sum_{l=1}^{N}r_{l}$.

##### Capacity

We capture and track the number of an individual’s familiar locations following the definition of Alessandretti et al..^35^ For each individual, we compute the location capacity *C* in each time window, normalized by the mean capacity of all users during the baseline period before the pandemic (January 3rd - March 7th, 2020).

Together with the capacity *C*, we also computed the number of locations added to (*A*) and deleted from (*D*) the set of familiar locations within a specific time interval and the previous time interval.^35^

#### Standard errors and statistical analysis

The errors associated with the mobility metrics in (Figure 2), (Figure 3), and (Figure 4) are estimated in terms of the standard errors of the median (and mean in the case of *Capacity* metric) computed across users. When computing errors for specific population groups standard errors are computed including only the users inferred to be part of that specific population group, e.g., *employed-unemployed* users, *Sex*, *Age*, *Income Race*, and *Education*.

Statistical analysis are carried out to test group-group mobility behaviour differences. In particular, we test the significance of the differences between demographic groups in (Figure 4) using Welch’s t-test.^46^ More specifically, to test the statistical difference in behaviour between the standardised unemployed population mobility and the employed population we use the one-sided t-test against 0 leveraging the scipy.stats.ttest_1samp python function. Similarly, to test the difference in behaviour between two unemployed population groups we use the unequal variance, i.e. two-sided Welch t-test, leveraging the the scipy.stats.ttest_ind python function. Results of the statistical tests are reported in Tables S3–S5.
