# Supplementary material for: Job loss disrupts individuals’ mobility and their exploratory patterns
Source: iScience. 2025 Jun 13;28(7):112892. doi: 10.1016/j.isci.2025.112892 (PMC12268674; doi:10.1016/j.isci.2025.112892)
Supplement: Document S1. Figures S1–S30, Tables S1–S5, and Methods S1–S6 [file mmc1.pdf]

iScience, Volume *28*

## Supplemental information

# Job loss disrupts individuals' mobility and their exploratory patterns

**Simone Centellegher, Marco De Nadai, Marco Tonin, Bruno Lepri, and Lorenzo Lucchini**

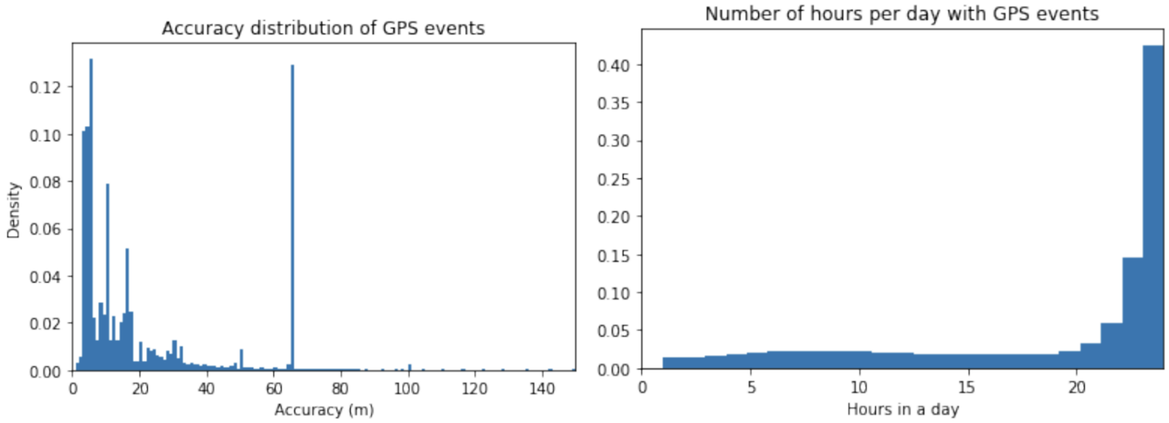


Figure S1: (left) Accuracy distribution of Cuebiq GPS events. (right) Distribution of the average number of hours (per user) covered by at least one GPS event.

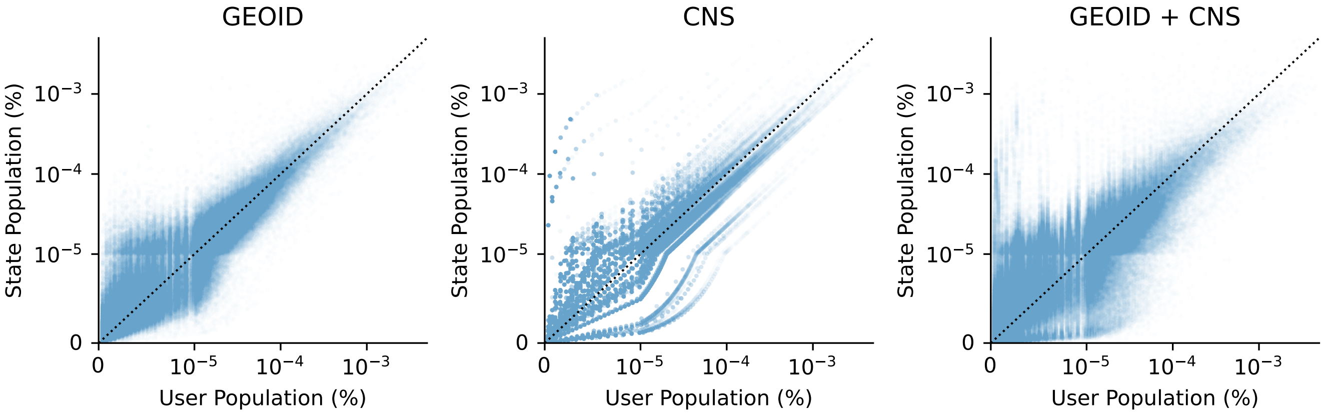


Figure S2: Comparision of Cuebiq vs Census population (each dot represents a mobile phone). The weights are shown to reflect the Census Block Group population (left), the industrial sector distribution (middle), and the combination of population and industrial sector distribution (right)

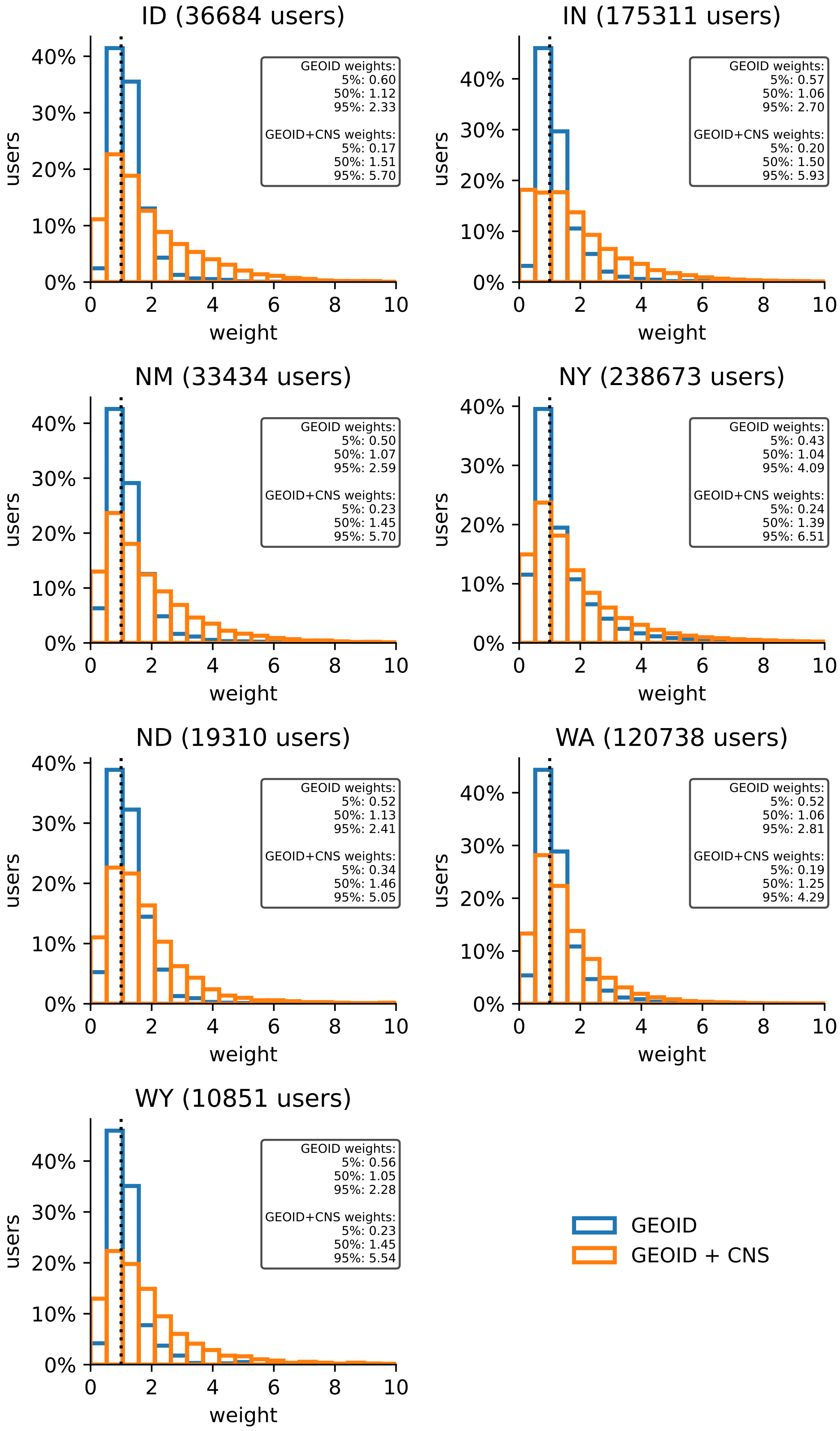


Figure S3: Weights' distribution by state. Each panel reports the weights' distribution for the seven states included in the study. In blue, the weights distribution accounting for GEOID (Census Block Group level) population representativeness only. In orange, the weights distribution accounting for both GEOID and the state-specific CNS workforce distribution.

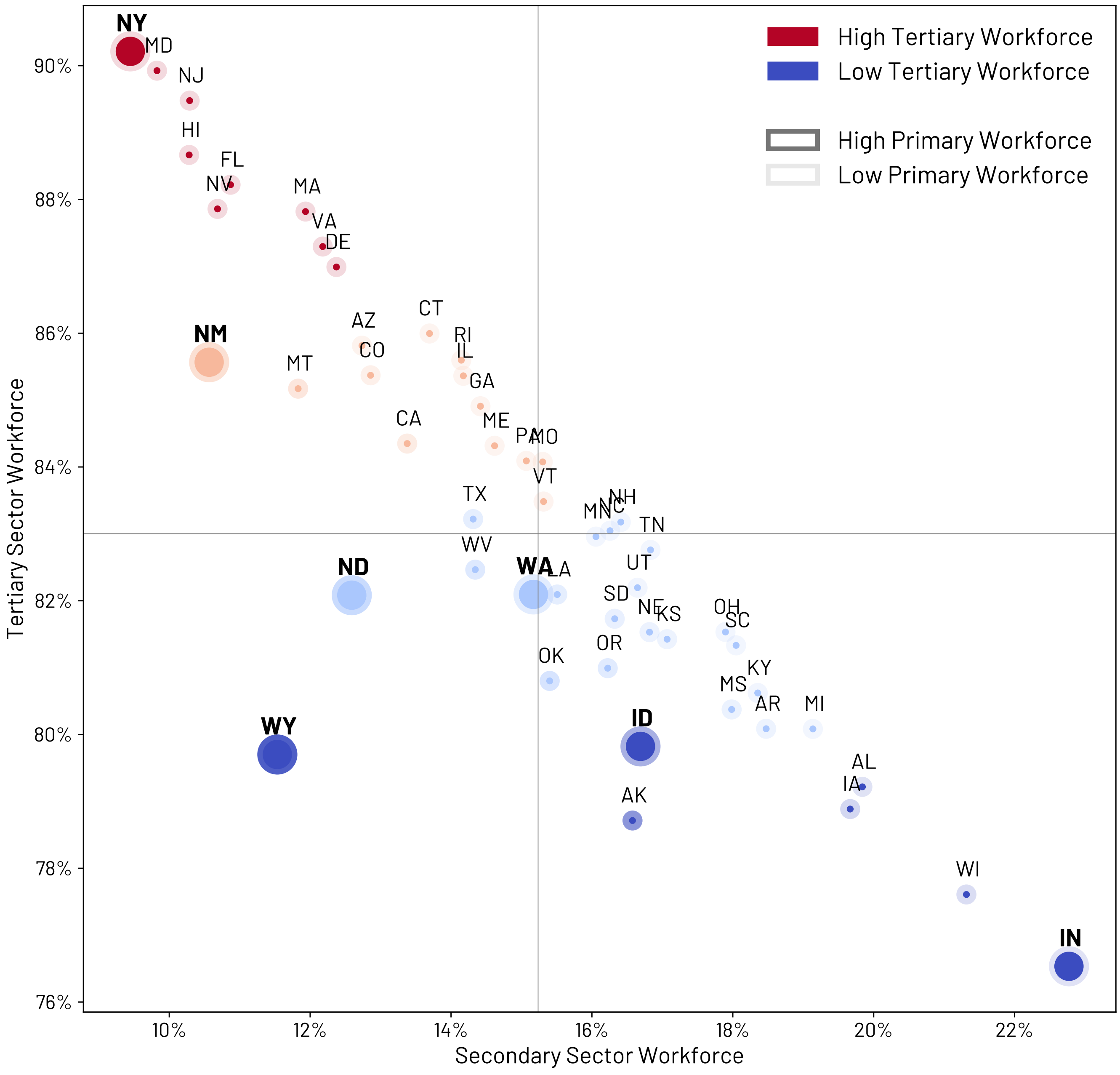


Figure S4: State selection based on the primary, secondary, and tertiary workforce composition in the US states. The selected states are New York (NY), Wyoming (WY), Indiana (IN), Idaho (ID), Washington (WA), North Dakota (ND), and New Mexico (NM). The internal colour code distinguishes states with a high-tertiary workforce (in red) from those with a low-tertiary workforce (in blue). The intensity of the shaded circle surrounding states highlights those states with higher levels of the primary workforce (higher intensity corresponds to higher primary workforce levels).

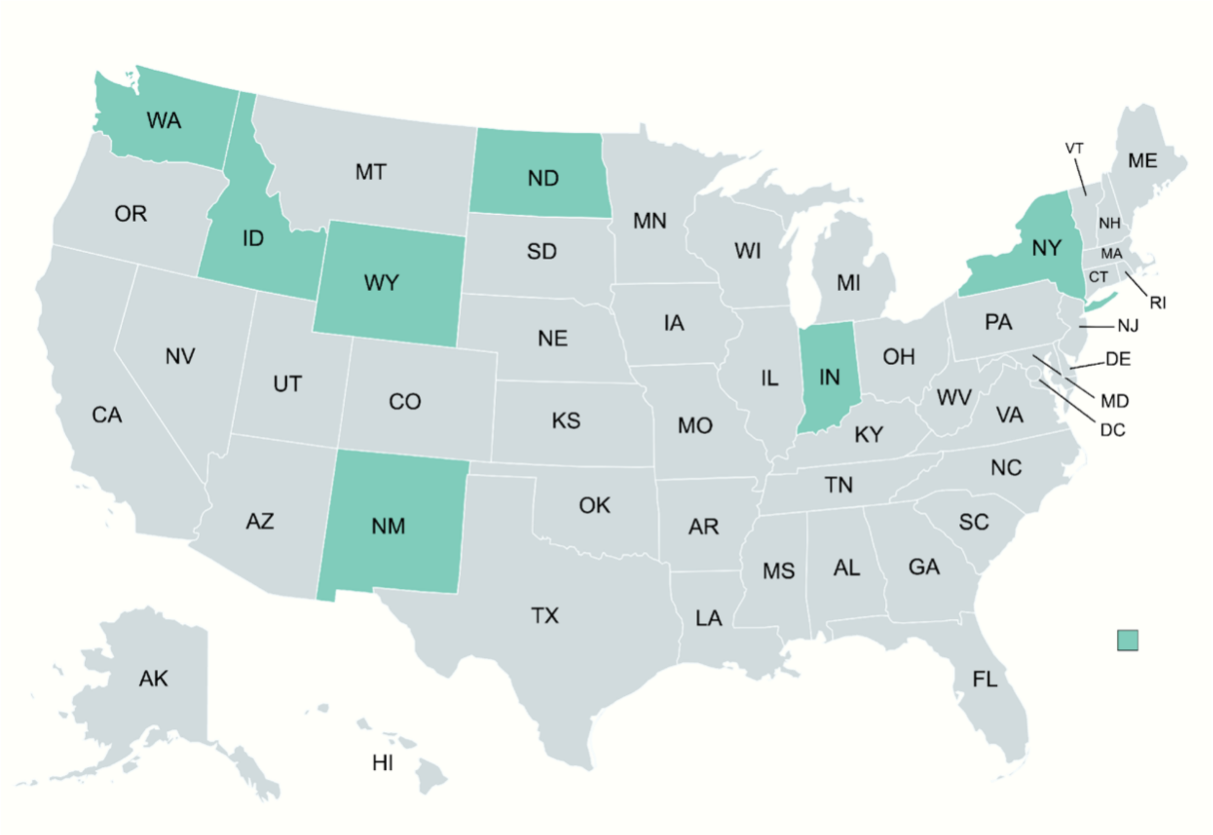


Figure S5: Geographical displacement of the countries included in this work analysis.

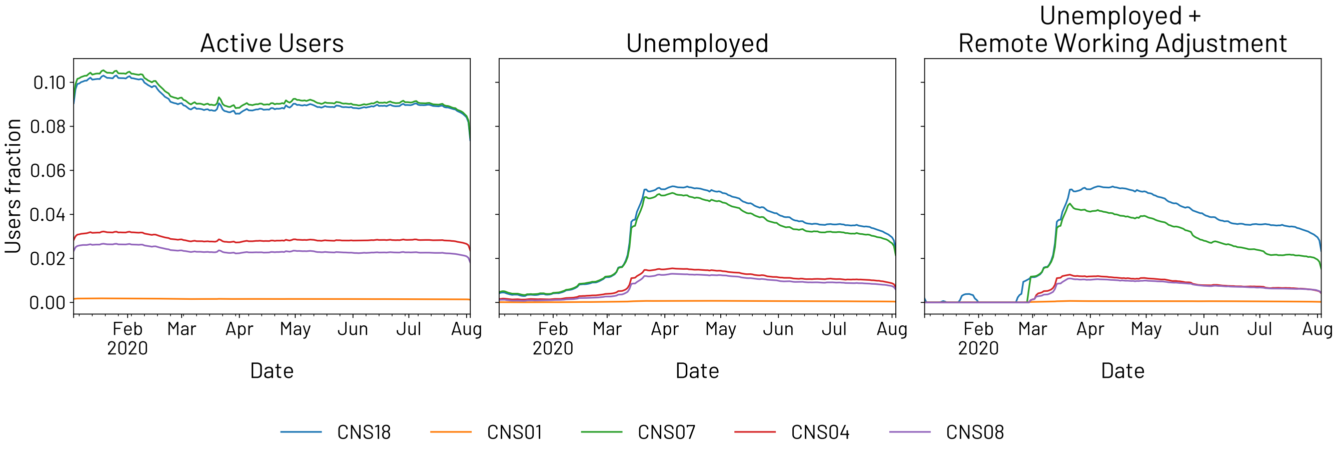


Figure S6: Remote working mechanism with NAICS sectors that are less teleworkable: Accommodation and Food Services (CNS18); Agriculture, Forestry, Fishing and Hunting (CNS01); Retail Trade (CNS07); Construction (CNS04); and Transportation and Warehousing (CNS08). Given the less remote workability, the adjustment leaves the unemployed unaffected (middle and left panel). The share of jobs that can be performed at home are reported in Tab. S1.

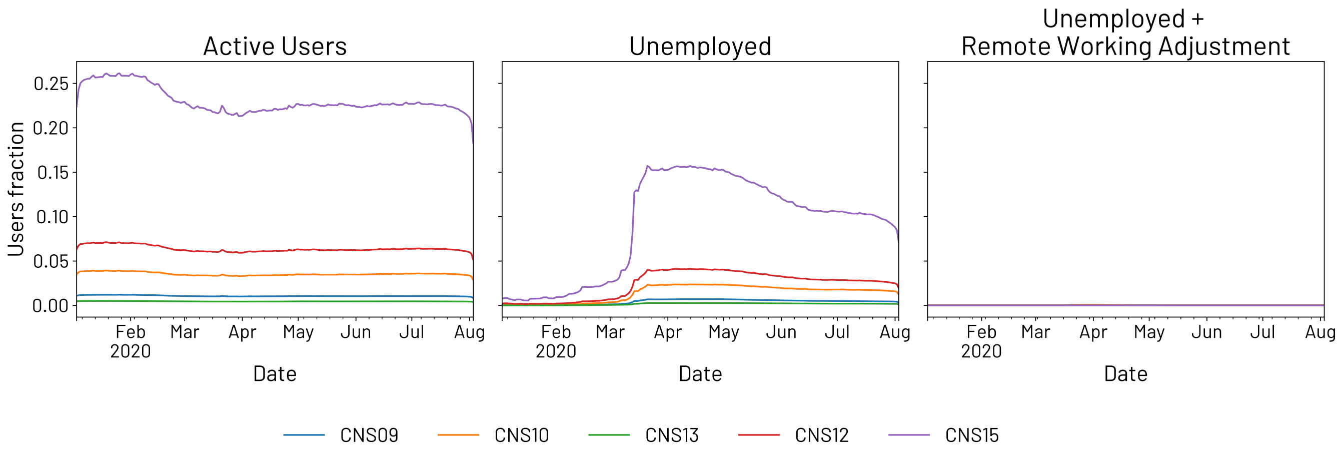


Figure S7: Remote working mechanism with NAICS sectors that are more teleworkable: Educational Services (CNS15); Professional, Scientific, and Technical Services (CNS12); Management of Companies and Enterprises (CNS13); Finance and Insurance (CNS10); and Information (CNS09). Given the more teleworkability the adjustment "removes" the unemployed since they are working from home (middle and left panel). The share of jobs that can be performed at home are reported in Tab. S1.

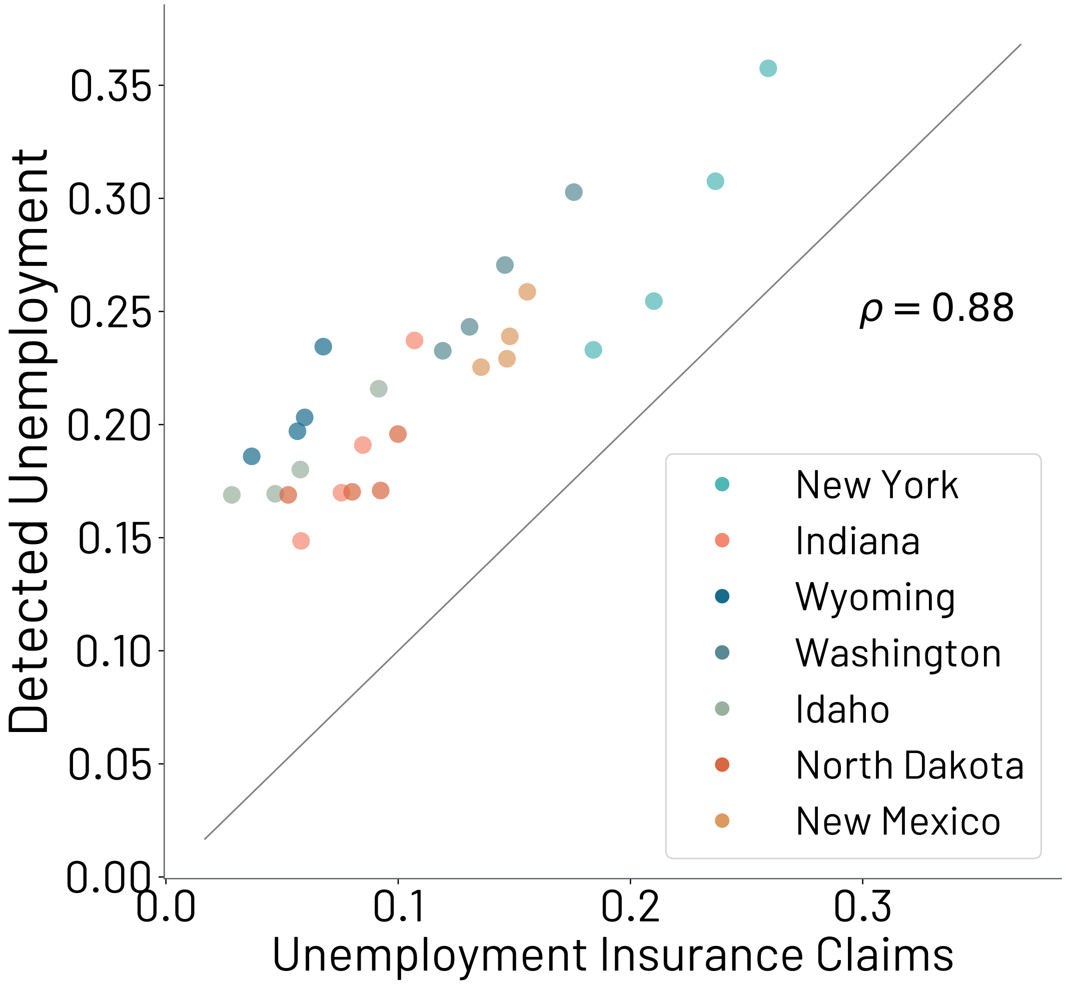


Figure S8: Algorithm evaluation and average Pearson correlation using the Unemployment Insurance (UI) claims data for each state and month.

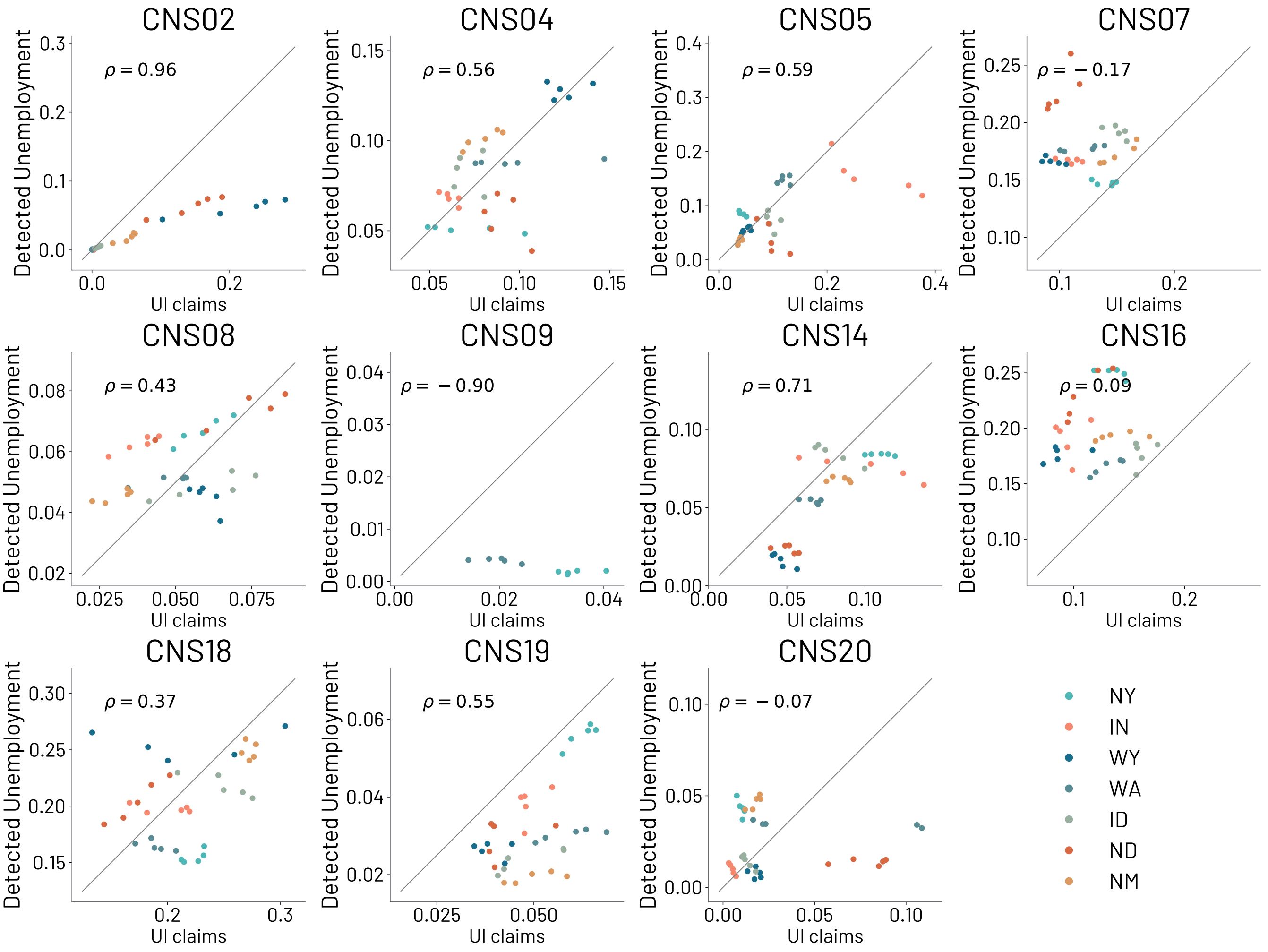


Figure S9: Algorithm evaluation and average Pearson correlation using the Unemployment Insurance (UI) claims data in each NAICS sector for each state and month. Only representative NACIS are showed.

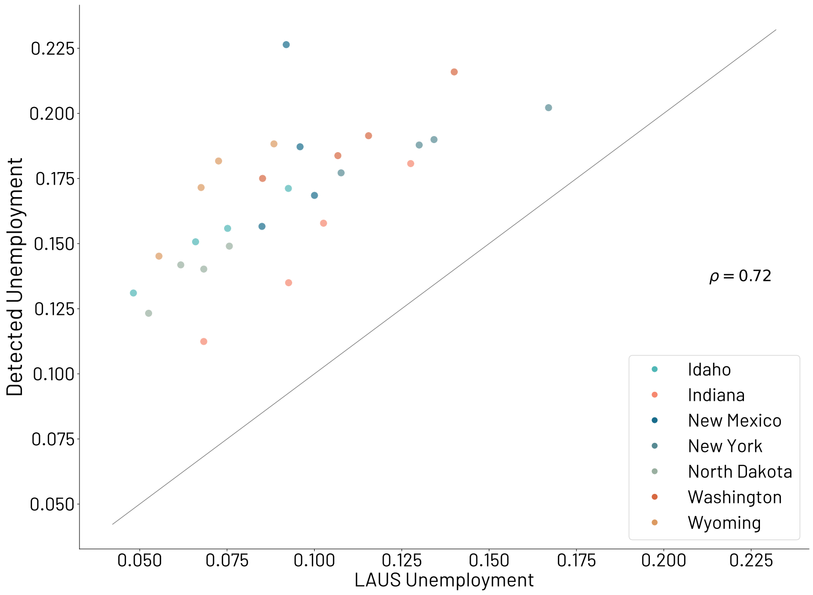


Figure S10: Algorithm evaluation and average Pearson correlation using the Local Area Unemployment Statistics (LAUS) data across all states and month.

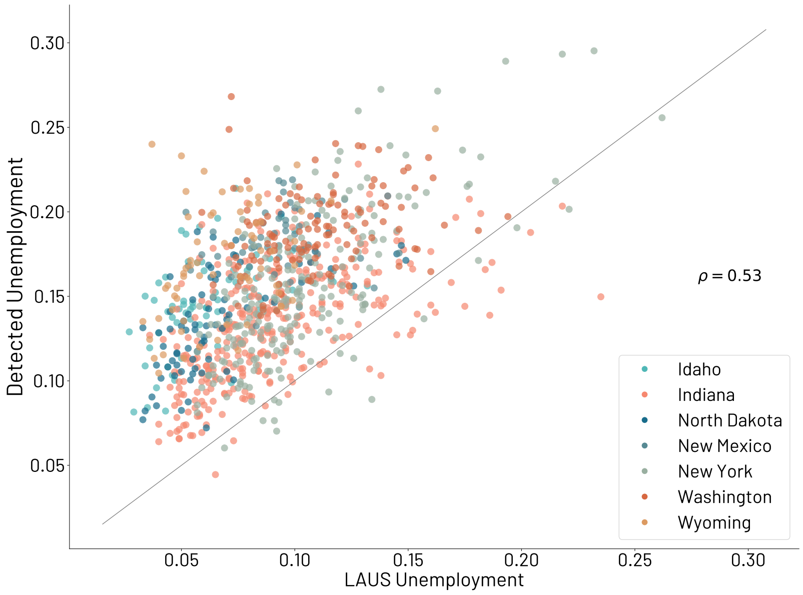


Figure S11: Algorithm performance evaluation and average Pearson correlation using Local Area Unemployment Statistics (LAUS) data across all counties and months.

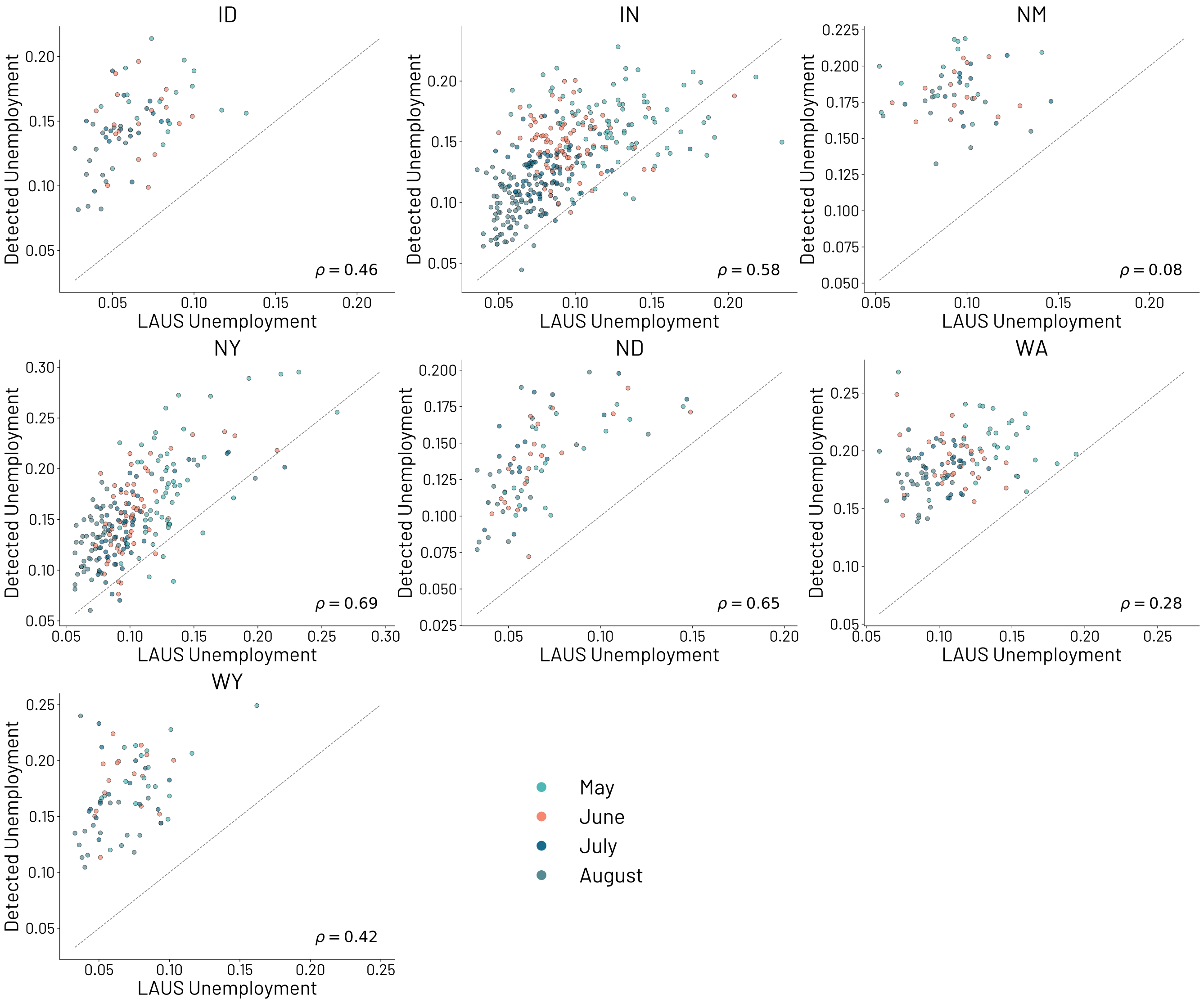


Figure S12: Algorithm performance evaluation and average Pearson correlation using Local Area Unemployment Statistics (LAUS) data across all counties divided by month.

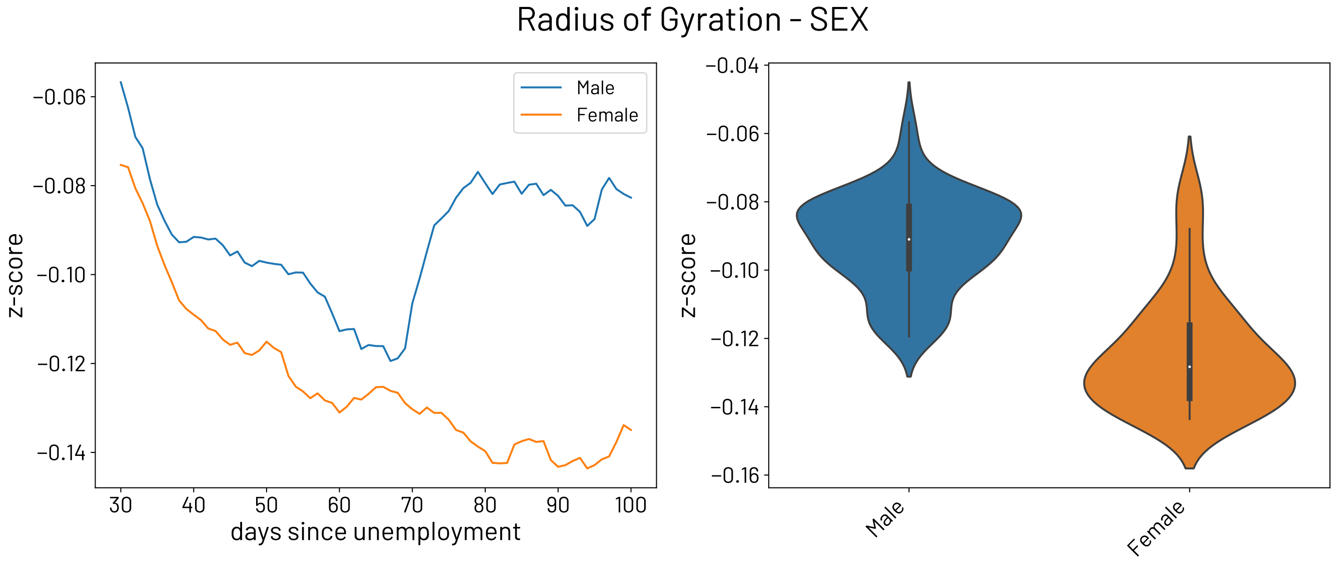


Figure S13: Z-scores for the radius of gyration divided by the Sex demographic indicator and their corresponding distribution.

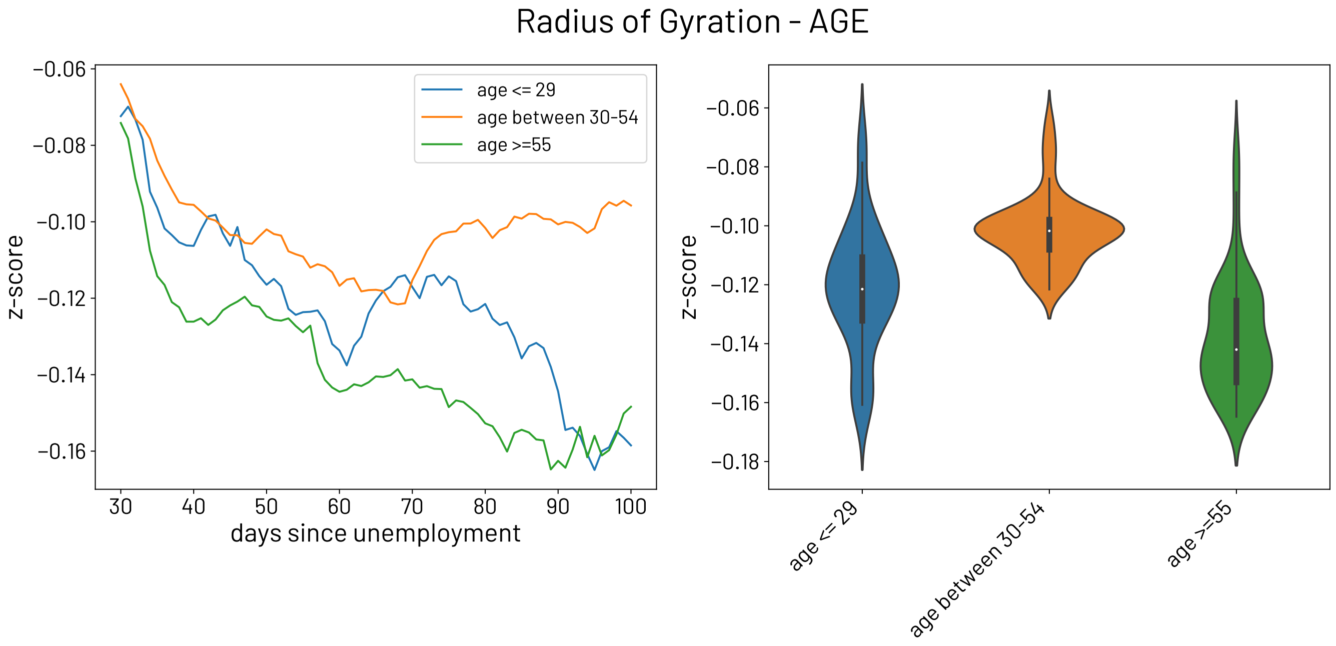


Figure S14: Z-scores for the radius of gyration divided by the Age demographic indicator and their corresponding distribution.

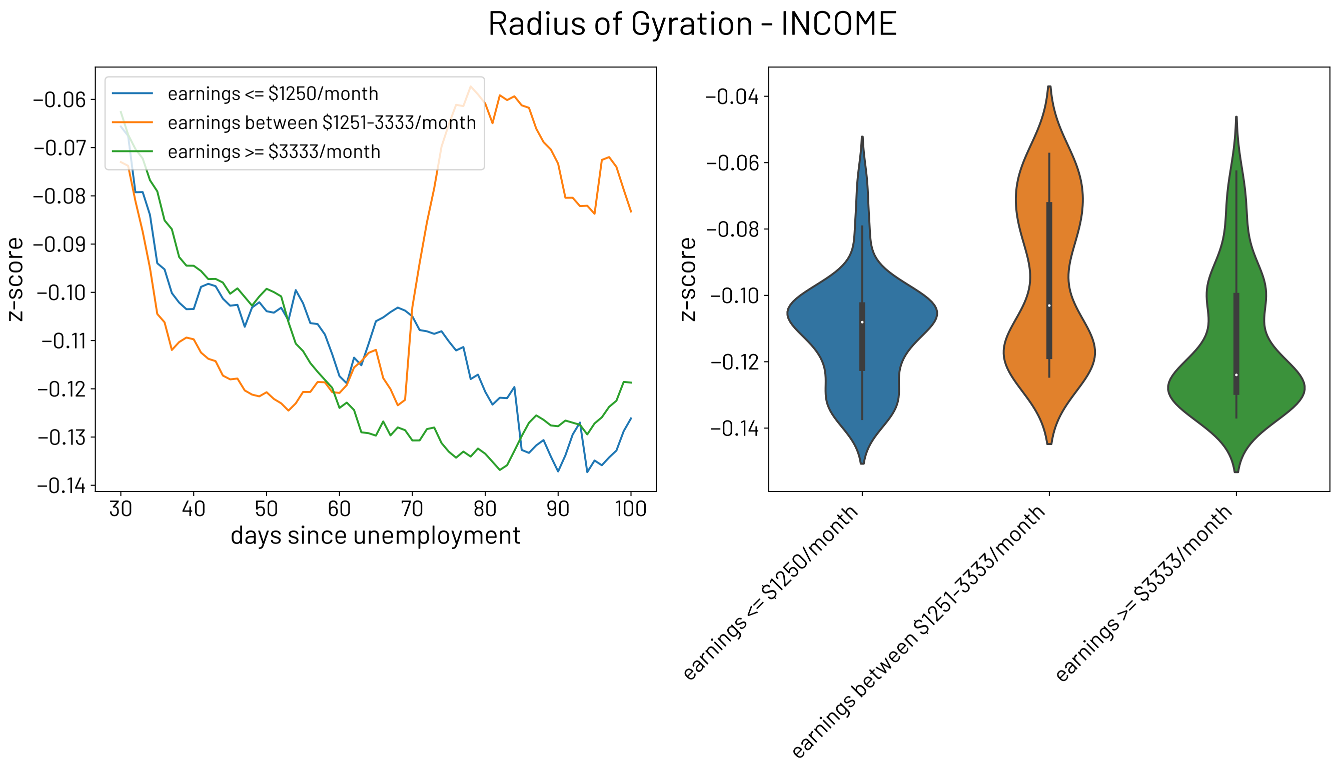


Figure S15: Z-scores for the radius of gyration divided by the Income demographic indicator and their corresponding distribution.

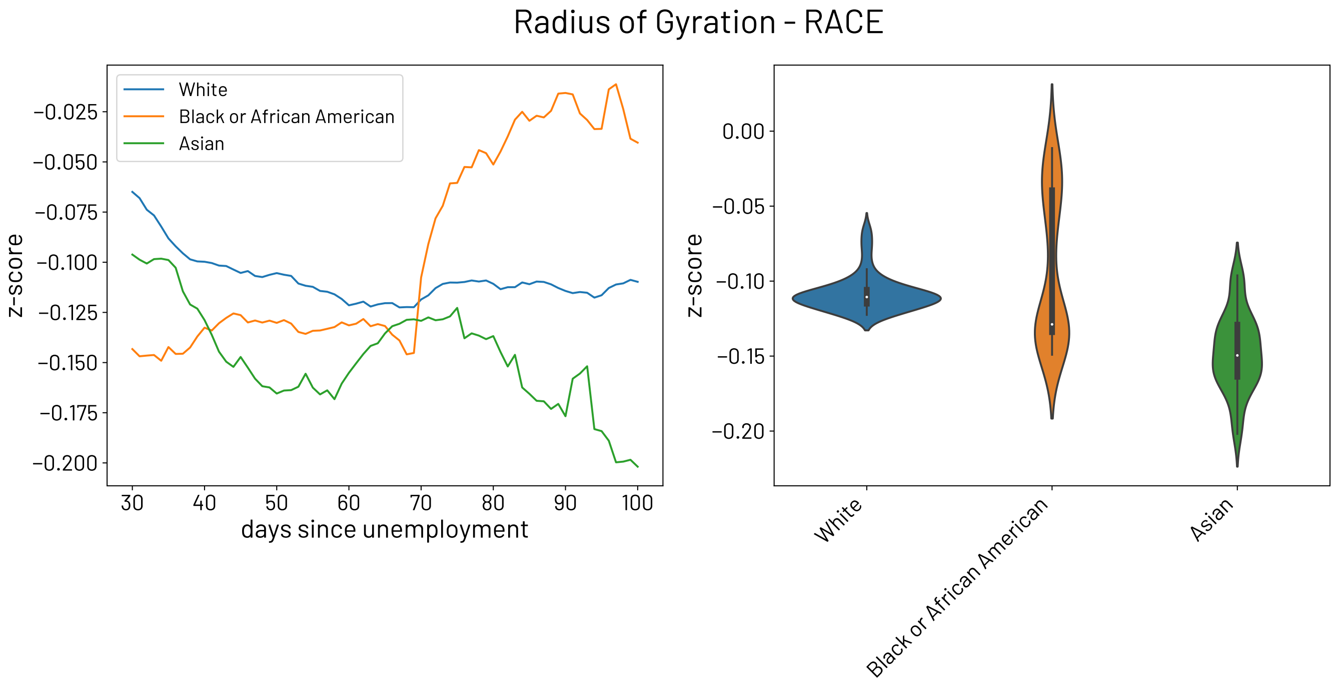


Figure S16: Z-scores for the radius of gyration divided by the Race demographic indicator and their corresponding distribution.

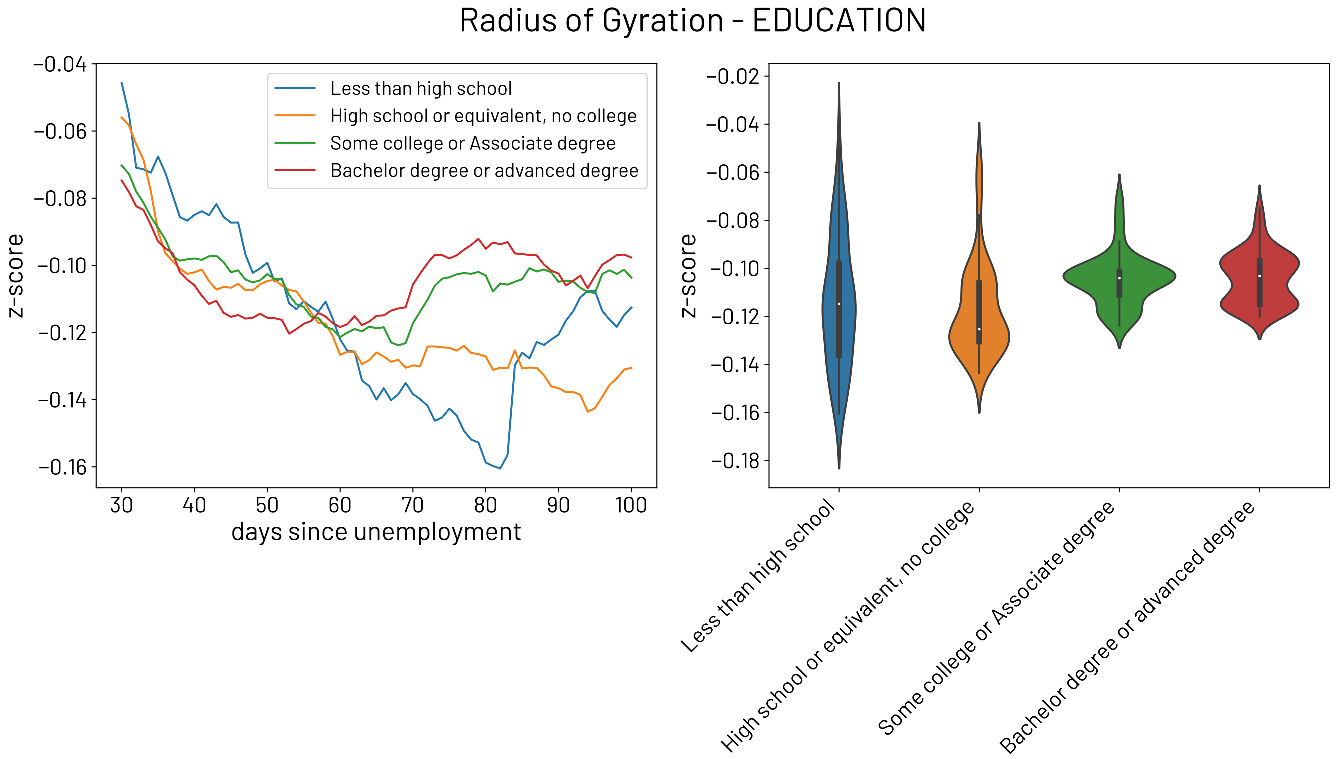


Figure S17: Z-scores for the radius of gyration divided by the Education demographic indicator and their corresponding distribution.

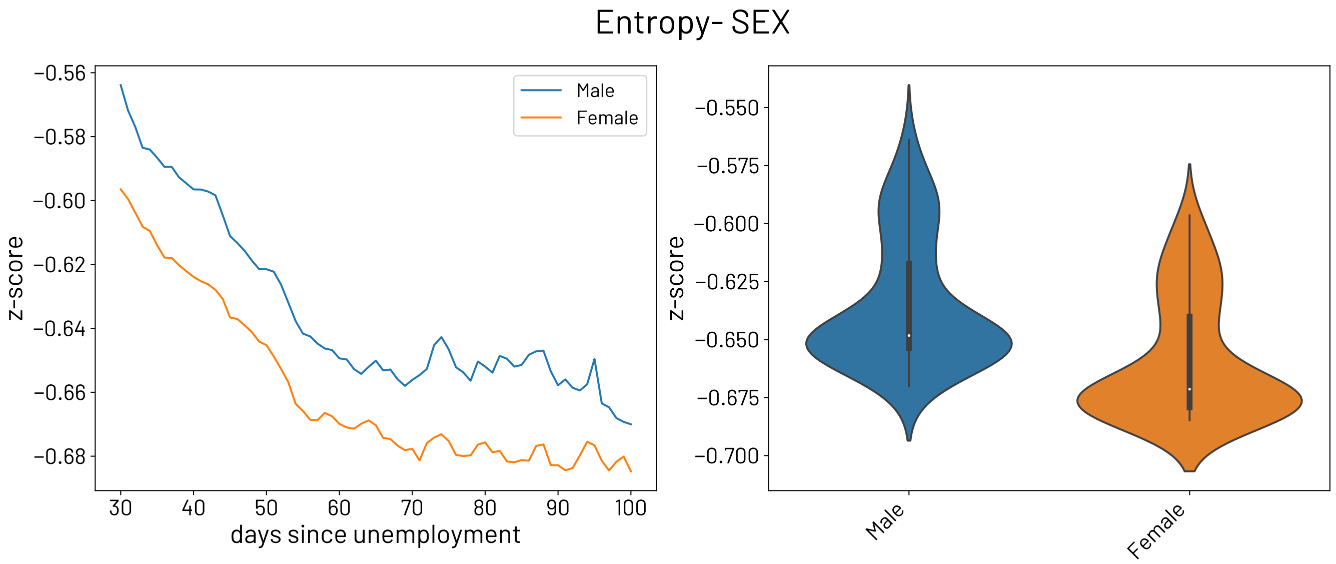


Figure S18: Z-scores for the entropy divided by the Sex demographic indicator and their corresponding distribution.

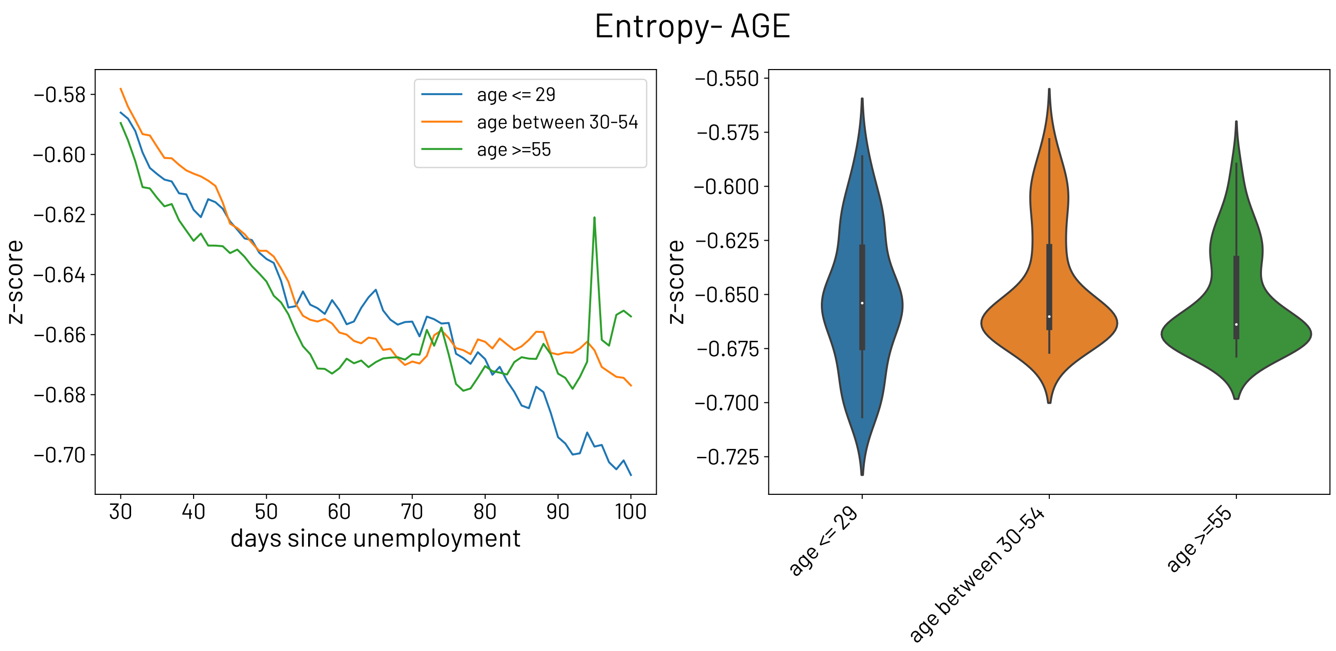


Figure S19: Z-scores for the entropy divided by the Age demographic indicator and their corresponding distribution.

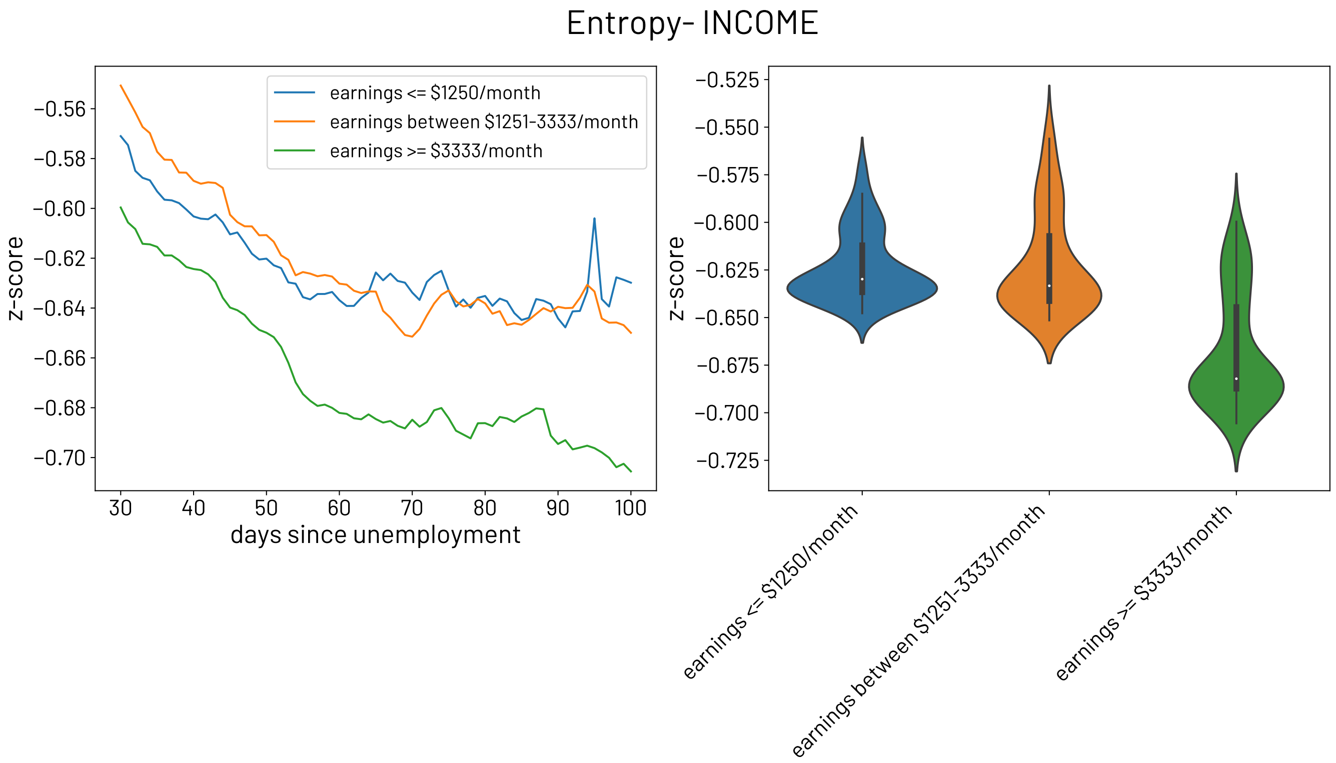


Figure S20: Z-scores for the entropy divided by the Income demographic indicator and their corresponding distribution.

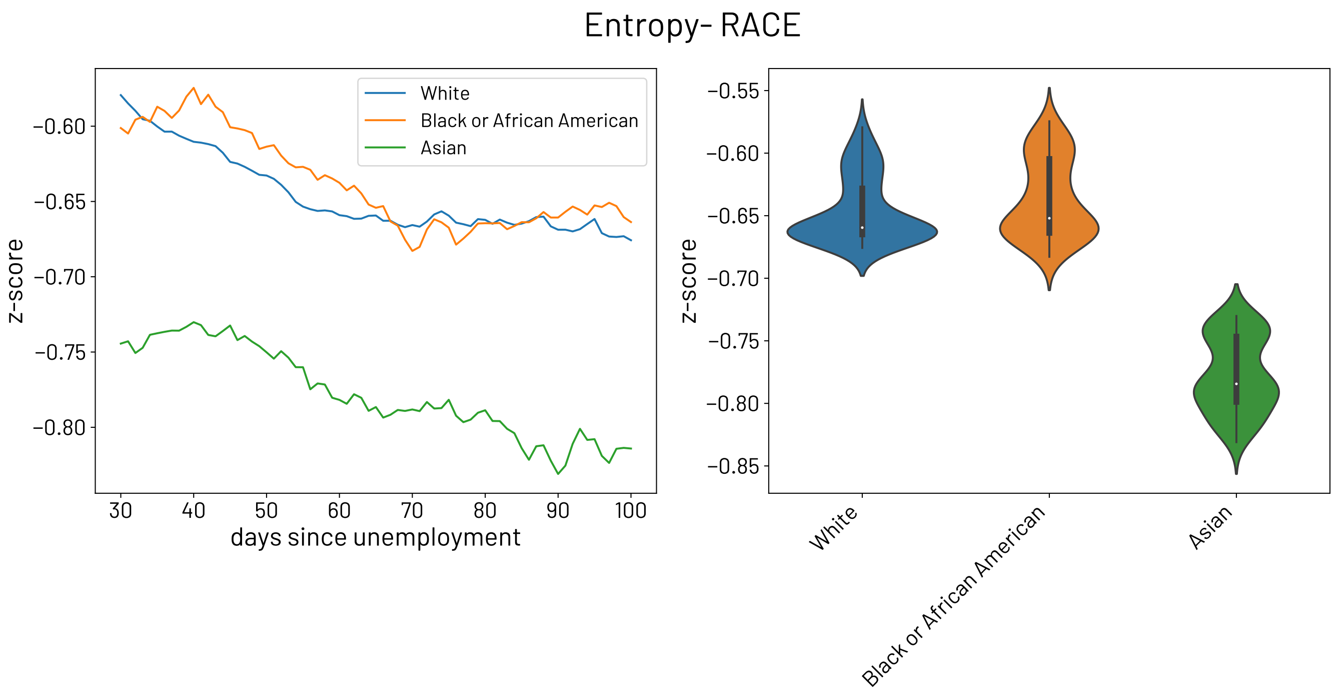


Figure S21: Z-scores for the entropy divided by the Race demographic indicator and their corresponding distribution.

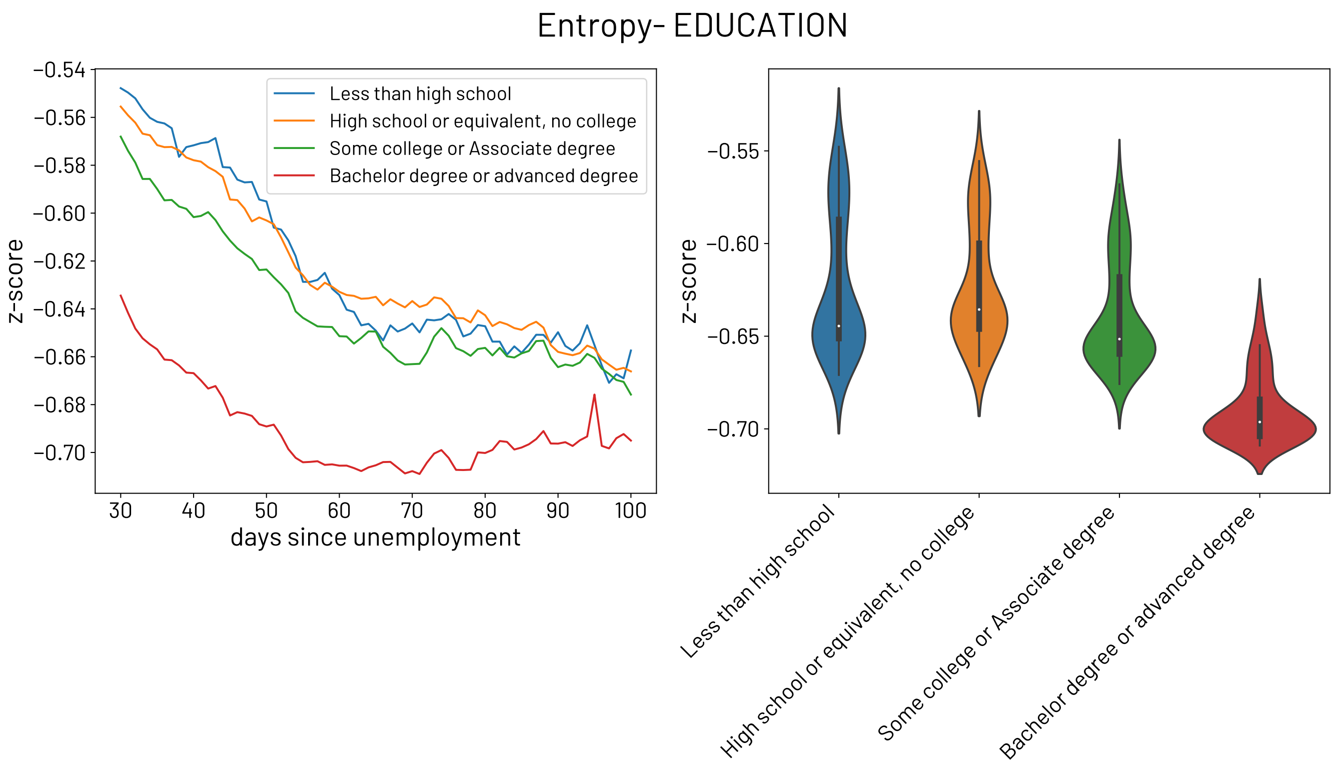


Figure S22: Z-scores for the entropy divided by the Education demographic indicator and their corresponding distribution.

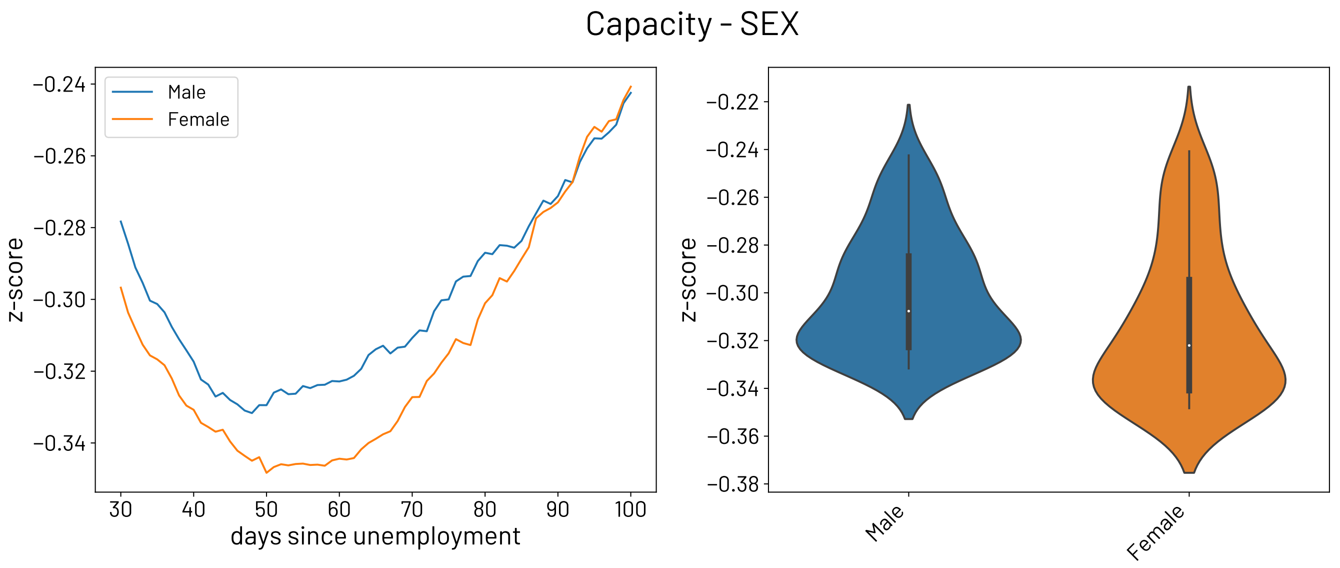


Figure S23: Z-scores for the capacity divided by the Sex demographic indicator and their corresponding distribution.

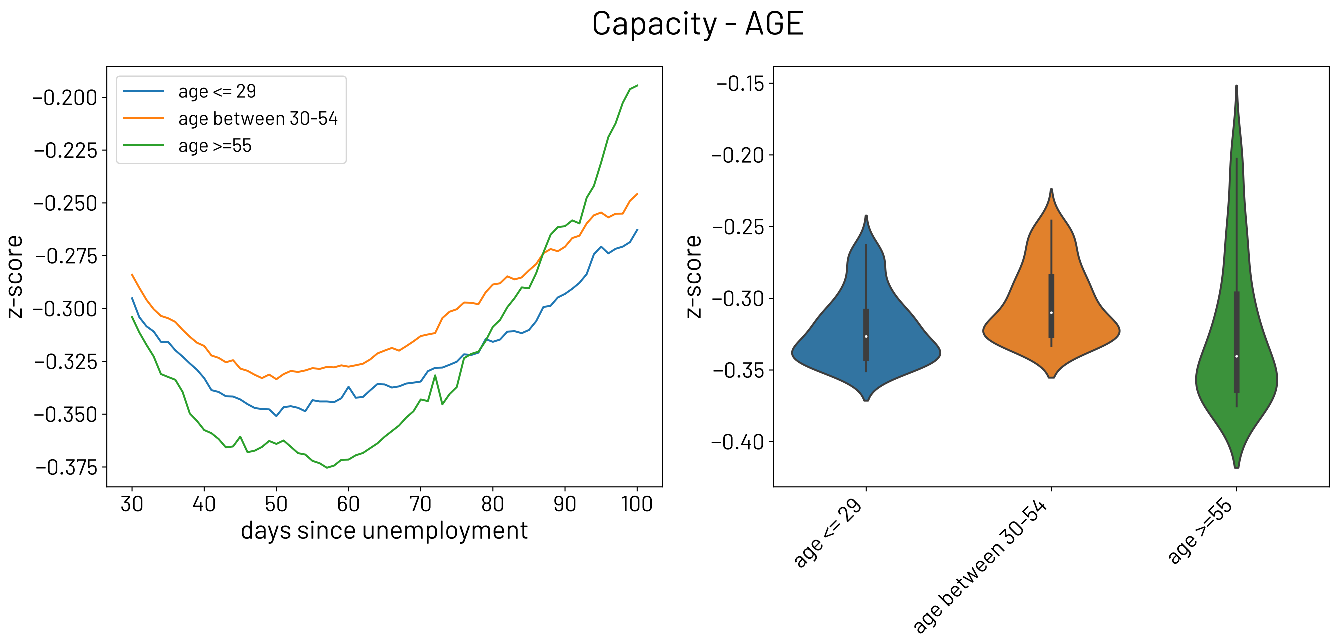


Figure S24: Z-scores for the capacity divided by the Age demographic indicator and their corresponding distribution.

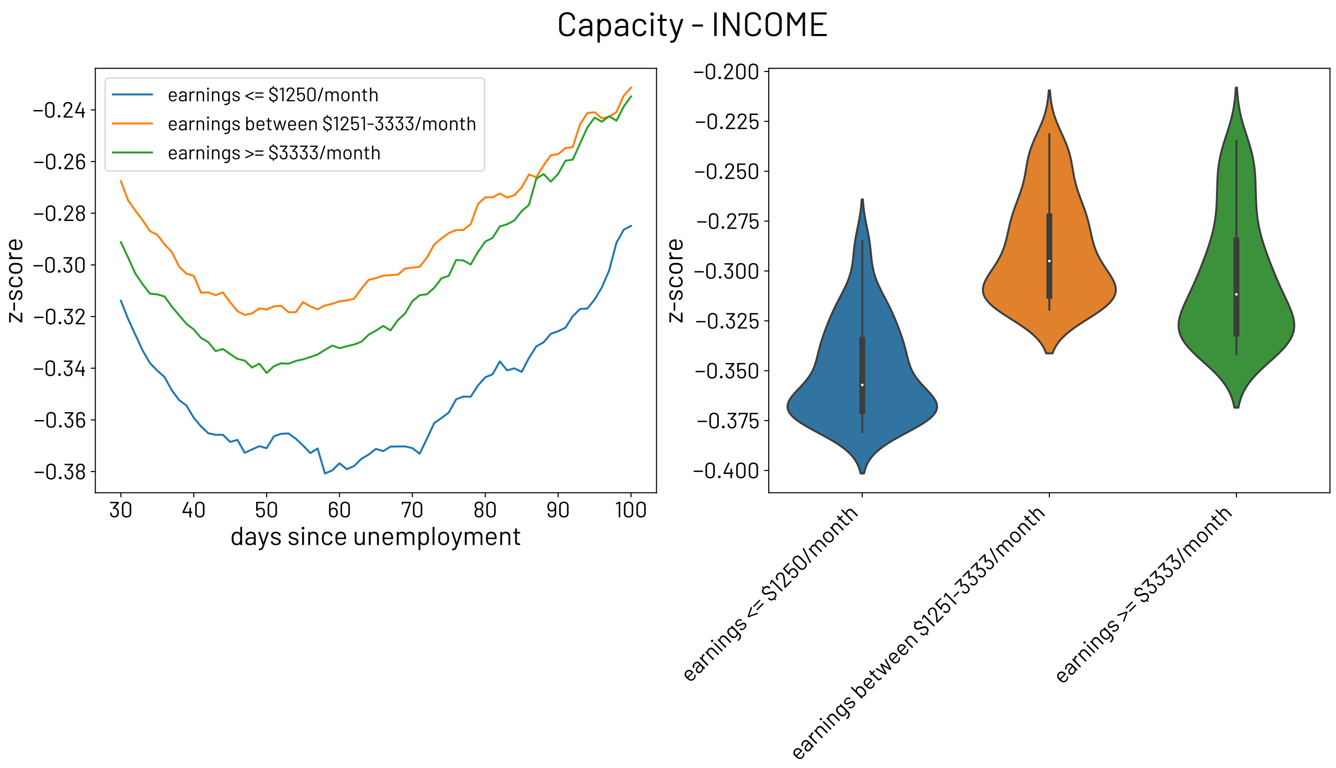


Figure S25: Z-scores for the capacity divided by the Income demographic indicator and their corresponding distribution.

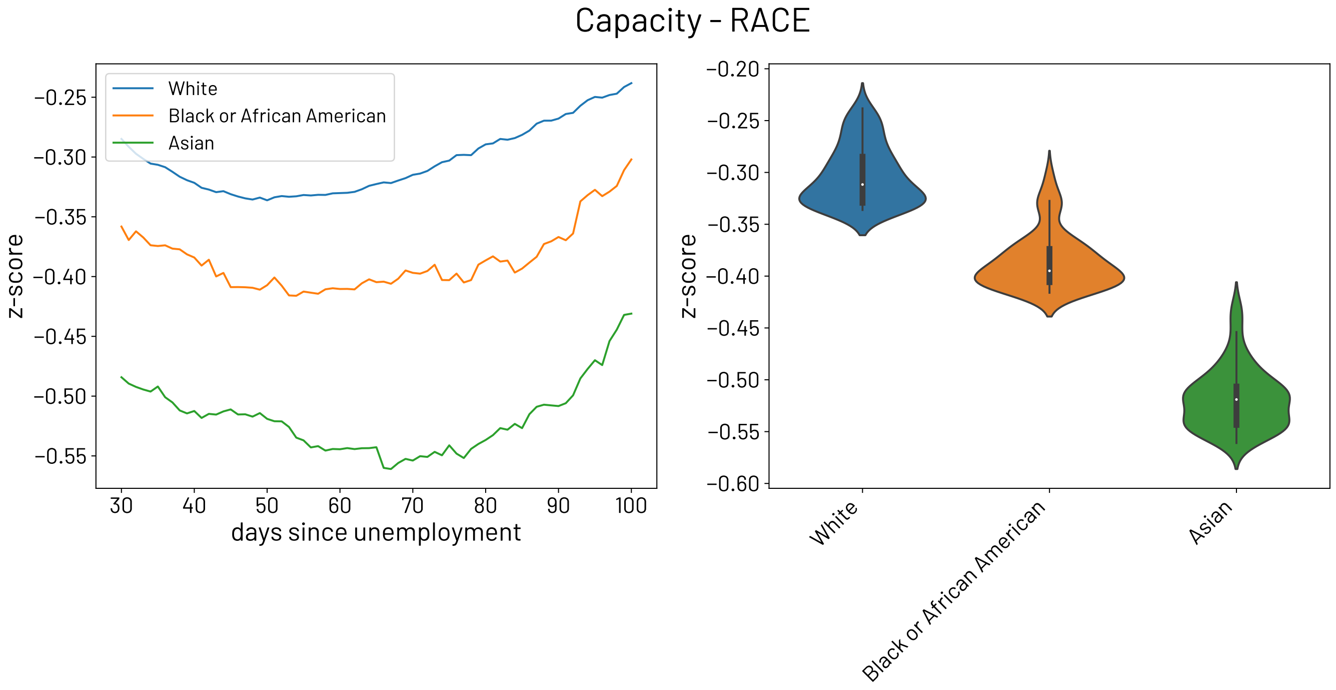


Figure S26: Z-scores for the capacity divided by the Race demographic indicator and their corresponding distribution.

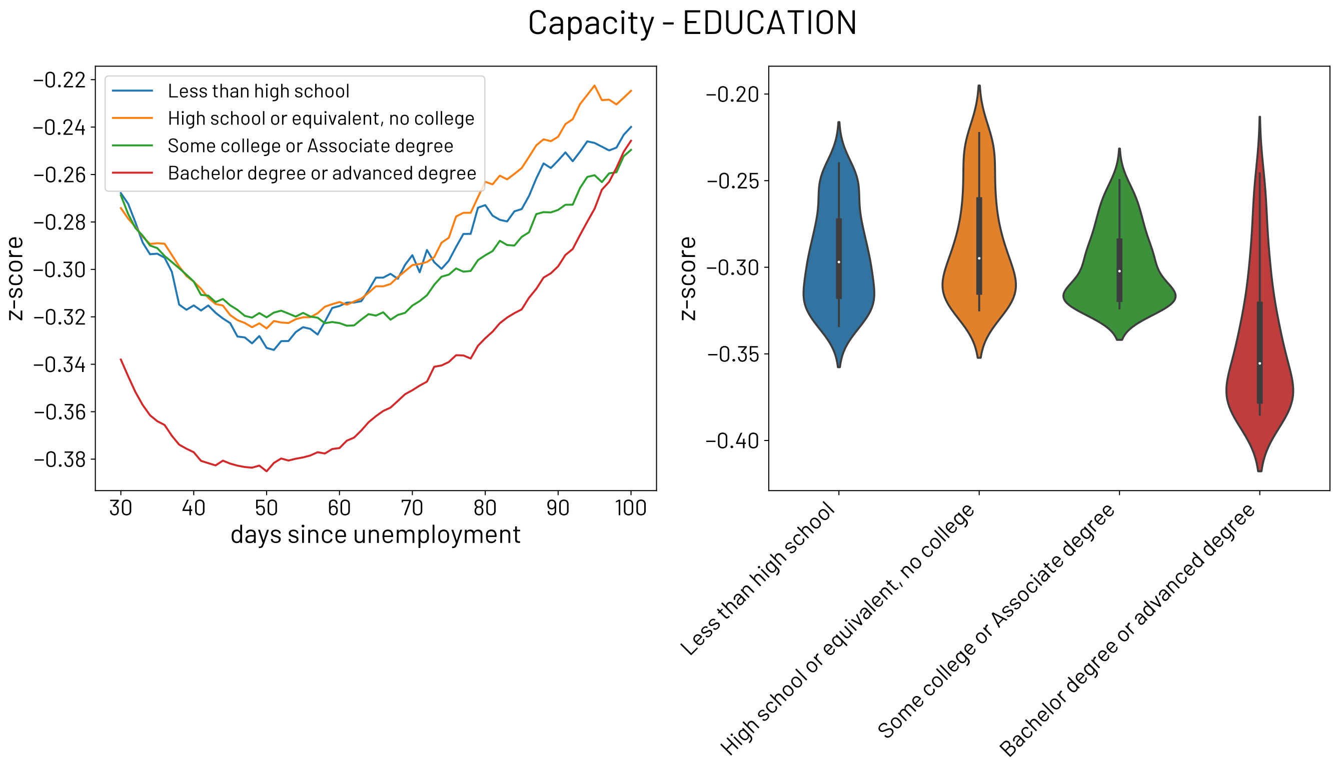


Figure S27: Z-scores for the capacity divided by the Education demographic indicator and their corresponding distribution.

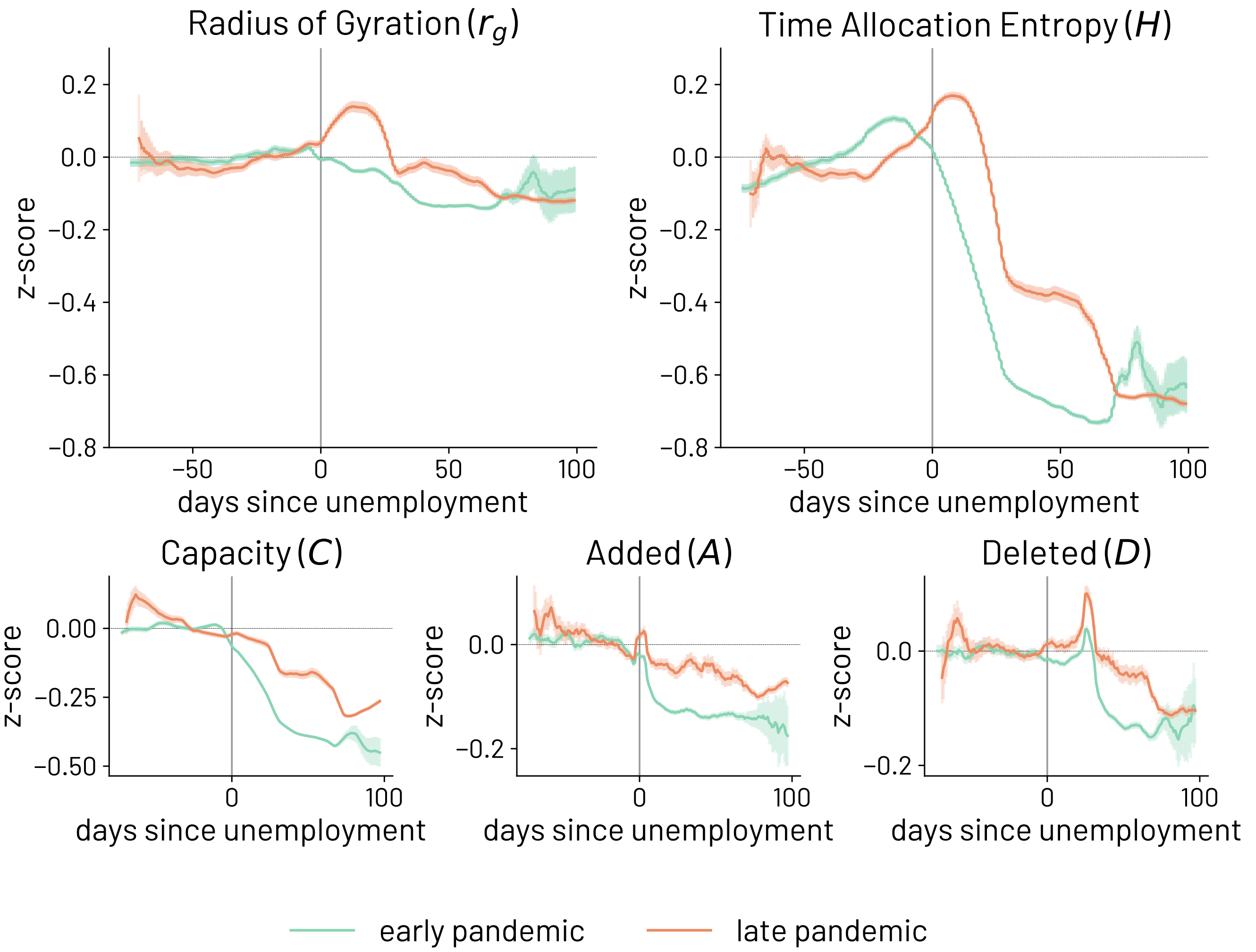


Figure S28: Early/late pandemic group-specific mobility since date of unemployment. The panels respectively report the radius of gyration, the time allocation entropy, the capacity, change in added locations, change in deleted locations for the groups of unemployed individual loosing their job during the early phase of the COVID-19 pandemic and in the later phase.

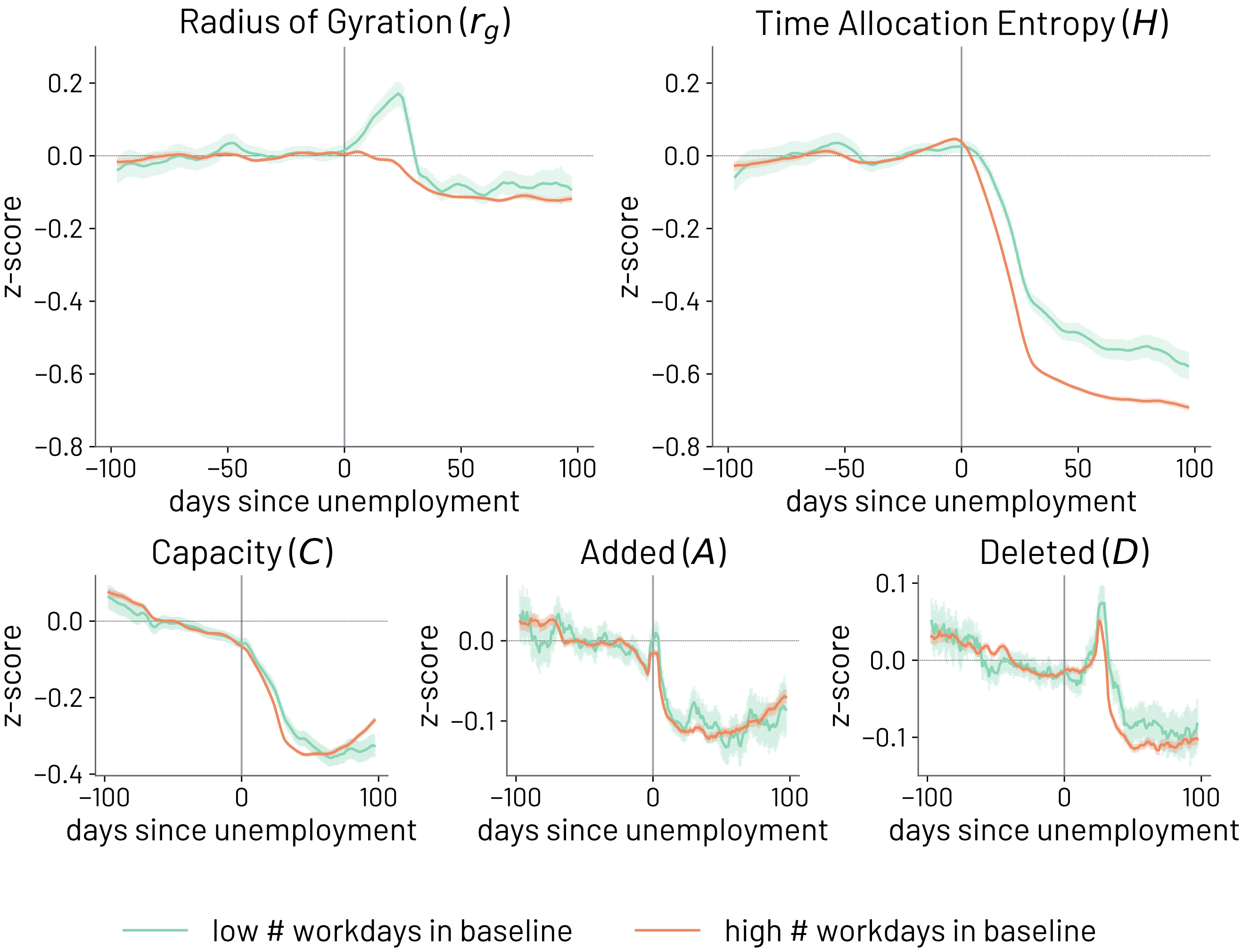


Figure S29: Group-specific mobility since date of unemployment: High/low work visits groups. The panels respectively report the radius of gyration, the time allocation entropy, the capacity, change in added locations, and change in deleted locations for the groups of unemployed individuals i) with a high frequency of work visits (in orange) and ii) low frequency of work visits during the baseline period.

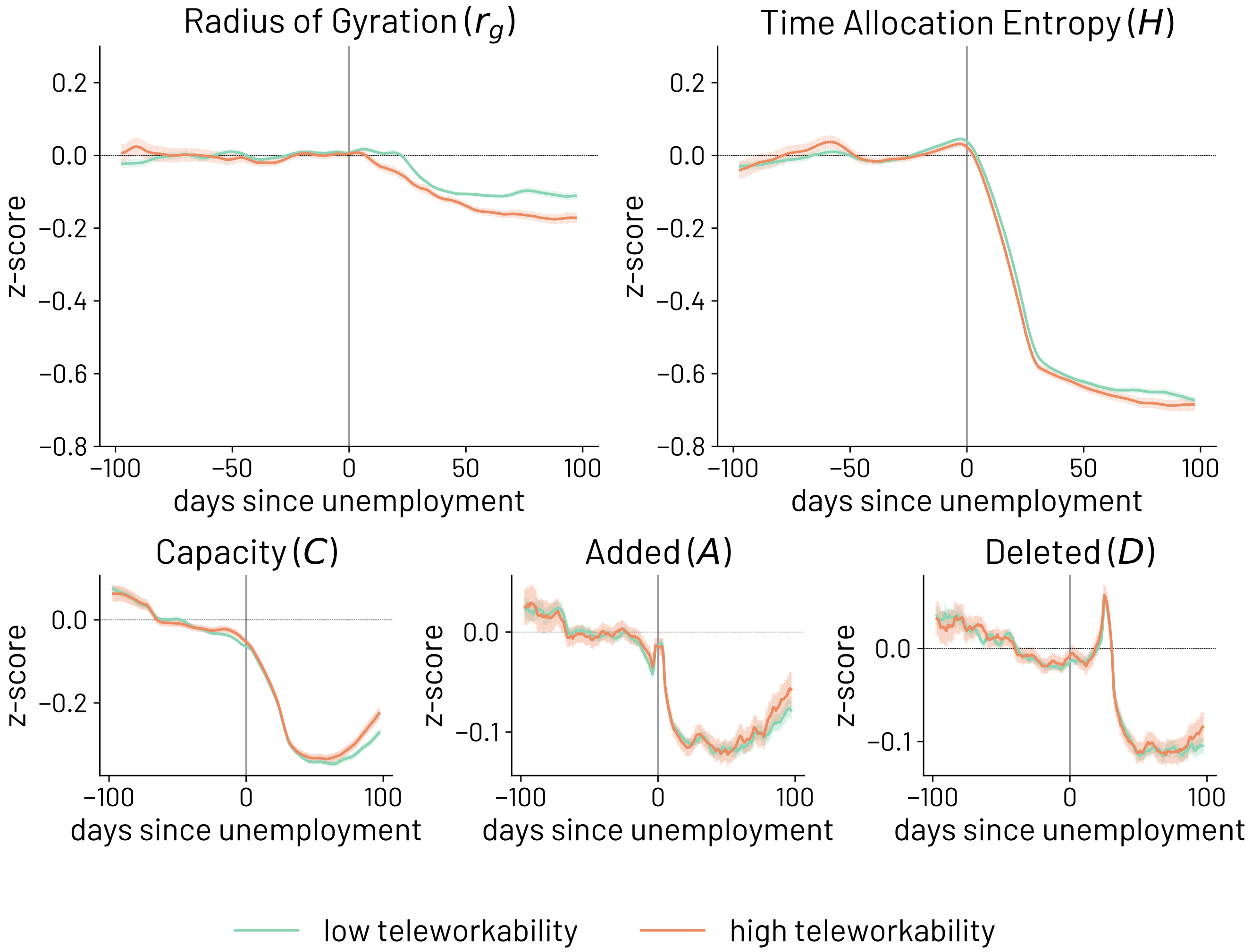


Figure S30: Group-specific mobility since date of unemployment: High/low teleworkability groups. The panels respectively report the radius of gyration, the time allocation entropy, the capacity, change in added locations, and change in deleted locations for the two groups of unemployed individuals i) individuals working in highly teleworkable sectors (in orange) and ii) individuals working in low teleworkability sectors.

| CNS | NAICS | Teleworkability |
|---|---|---|
| CNS01 | Agriculture, Forestry, Fishing and Hunting | 0.076394 |
| CNS02 | Mining, Quarrying, and Oil and Gas Extraction | 0.254480 |
| CNS03 | Utilities | 0.370015 |
| CNS04 | Construction | 0.185599 |
| CNS05 | Manufacturing | 0.224803 |
| CNS06 | Wholesale Trade | 0.517553 |
| CNS07 | Retail Trade | 0.143435 |
| CNS08 | Transportation and Warehousing | 0.186145 |
| CNS09 | Information | 0.717062 |
| CNS10 | Finance and Insurance | 0.762030 |
| CNS11 | Real Estate and Rental and Leasing | 0.418109 |
| CNS12 | Professional, Scientific, and Technical Services | 0.802757 |
| CNS13 | Management of Companies and Enterprises | 0.791891 |
| CNS14 | Administrative and Support and Waste Managemen... | 0.310632 |
| CNS15 | Educational Services | 0.826465 |
| CNS16 | Health Care and Social Assistance | 0.252522 |
| CNS17 | Arts, Entertainment, and Recreation | 0.297494 |
| CNS18 | Accommodation and Food Services | 0.035366 |
| CNS19 | Other Services (except Public Administration) | 0.312351 |
| CNS20 | Federal, State, and Local Government, excludin... | 0.414776 |

Table S1: Share of jobs that can be performed at home divided by their respective NAICS sector.

| NAICS sector | Description | Economic sector |
|---|---|---|
| 11 | Agriculture, Forestry, Fishing and Hunting | Primary |
| 21 | Mining, Quarrying, and Oil and Gas Extraction | Primary |
| 22 | Utilities | Secondary |
| 23 | Construction | Secondary |
| 31-33 | Manufacturing | Secondary |
| 42 | Wholesale Trade | Tertiary |
| 44-45 | Retail Trade | Tertiary |
| 48-49 | Transportation and Warehousing | Tertiary |
| 51 | Information | Tertiary |
| 52 | Finance and Insurance | Tertiary |
| 53 | Real Estate and Rental and Leasing | Tertiary |
| 54 | Professional, Scientific, and Technical Services | Tertiary |
| 55 | Management of Companies and Enterprises | Tertiary |
| 56 | Administrative and Support and Waste Management and Remediation Services | Tertiary |
| 61 | Educational Services | Tertiary |
| 62 | Health Care and Social Assistance | Tertiary |
| 71 | Arts, Entertainment, and Recreation | Tertiary |
| 72 | Accommodation and Food Services | Tertiary |
| 81 | Other Services (except Public Administration) | Tertiary |
| 92 | Public Administration | Tertiary |

Table S2: NAICS sectors and their corresponding economic sectors.

| Demographic | Mobility Metric | Group | Group avg | T-test | p-value | Significance |
|---|---|---|---|---|---|---|
| SEX | Radius of gyration | Male | -0.08 | -4.64e+01 | 0.00e+00 | *** |
| SEX | Radius of gyration | Female | -0.11 | -6.43e+01 | 0.00e+00 | *** |
| SEX | Entropy | Male | -0.55 | -2.55e+02 | 0.00e+00 | *** |
| SEX | Entropy | Female | -0.6 | -2.81e+02 | 0.00e+00 | *** |
| SEX | Capacity | Male | -0.3 | -1.62e+02 | 0.00e+00 | *** |
| SEX | Capacity | Female | -0.31 | -1.61e+02 | 0.00e+00 | *** |
| AGE | Radius of gyration | age <= 29 | -0.1 | -3.03e+01 | 1.76e-199 | *** |
| AGE | Radius of gyration | age between 30-54 | -0.09 | -6.22e+01 | 0.00e+00 | *** |
| AGE | Radius of gyration | age >=55 | -0.11 | -3.64e+01 | 1.73e-285 | *** |
| AGE | Entropy | age <= 29 | -0.57 | -1.36e+02 | 0.00e+00 | *** |
| AGE | Entropy | age between 30-54 | -0.57 | -3.25e+02 | 0.00e+00 | *** |
| AGE | Entropy | age >=55 | -0.57 | -1.34e+02 | 0.00e+00 | *** |
| AGE | Capacity | age <= 29 | -0.31 | -8.58e+01 | 0.00e+00 | *** |
| AGE | Capacity | age between 30-54 | -0.3 | -1.93e+02 | 0.00e+00 | *** |
| AGE | Capacity | age >=55 | -0.32 | -8.87e+01 | 0.00e+00 | *** |
| INCOME | Radius of gyration | earnings <= $1250/month | -0.09 | -3.06e+01 | 2.96e-203 | *** |
| INCOME | Radius of gyration | earnings between $1251-3333/month | -0.1 | -4.70e+01 | 0.00e+00 | *** |
| INCOME | Radius of gyration | earnings >= $3333/month | -0.09 | -5.34e+01 | 0.00e+00 | *** |
| INCOME | Entropy | earnings <= $1250/month | -0.55 | -1.46e+02 | 0.00e+00 | *** |
| INCOME | Entropy | earnings between $1251-3333/month | -0.56 | -1.98e+02 | 0.00e+00 | *** |
| INCOME | Entropy | earnings >= $3333/month | -0.58 | -2.87e+02 | 0.00e+00 | *** |
| INCOME | Capacity | earnings <= $1250/month | -0.34 | -1.08e+02 | 0.00e+00 | *** |
| INCOME | Capacity | earnings between $1251-3333/month | -0.3 | -1.21e+02 | 0.00e+00 | *** |
| INCOME | Capacity | earnings >= $3333/month | -0.3 | -1.63e+02 | 0.00e+00 | *** |
| RACE | Radius of gyration | White | -0.09 | -7.56e+01 | 0.00e+00 | *** |
| RACE | Radius of gyration | Black or African American | -0.11 | -9.67e+00 | 7.42e-22 | *** |
| RACE | Radius of gyration | Asian | -0.15 | -1.43e+01 | 4.27e-44 | *** |
| RACE | Entropy | White | -0.57 | -3.70e+02 | 0.00e+00 | *** |
| RACE | Entropy | Black or African American | -0.62 | -4.59e+01 | 0.00e+00 | *** |
| RACE | Entropy | Asian | -0.75 | -4.93e+01 | 0.00e+00 | *** |
| RACE | Capacity | White | -0.3 | -2.23e+02 | 0.00e+00 | *** |
| RACE | Capacity | Black or African American | -0.39 | -3.92e+01 | 5.31e-297 | *** |
| RACE | Capacity | Asian | -0.51 | -4.53e+01 | 0.00e+00 | *** |
| EDUCATION | Radius of gyration | Less than high school | -0.1 | -2.04e+01 | 7.08e-92 | *** |
| EDUCATION | Radius of gyration | High school or equivalent, no college | -0.09 | -4.01e+01 | 0.00e+00 | *** |
| EDUCATION | Radius of gyration | Some college or Associate degree | -0.09 | -4.74e+01 | 0.00e+00 | *** |
| EDUCATION | Radius of gyration | Bachelor degree or advanced degree | -0.1 | -4.17e+01 | 0.00e+00 | *** |
| EDUCATION | Entropy | Less than high school | -0.55 | -8.76e+01 | 0.00e+00 | *** |
| EDUCATION | Entropy | High school or equivalent, no college | -0.54 | -1.88e+02 | 0.00e+00 | *** |
| EDUCATION | Entropy | Some college or Associate degree | -0.56 | -2.30e+02 | 0.00e+00 | *** |
| EDUCATION | Entropy | Bachelor degree or advanced degree | -0.62 | -2.22e+02 | 0.00e+00 | *** |
| EDUCATION | Capacity | Less than high school | -0.3 | -5.52e+01 | 0.00e+00 | *** |
| EDUCATION | Capacity | High school or equivalent, no college | -0.3 | -1.23e+02 | 0.00e+00 | *** |
| EDUCATION | Capacity | Some college or Associate degree | -0.3 | -1.36e+02 | 0.00e+00 | *** |
| EDUCATION | Capacity | Bachelor degree or advanced degree | -0.33 | -1.26e+02 | 0.00e+00 | *** |

Table S3: Comparison of the groups of a socio-demographic factor against the entire employed population. The statistical significance is computed with the T-test statistic. Statistical significance is reported using the following notation for different p-values: * ($p < 0.05$), ** ($p < 0.01$), *** ($p < 0.001$), $ns$ ($p >= 0.05$).

| Demographic | Mobility Metric | Group1 | Group2 | Welch t-test | p-value | Significant |
|---|---|---|---|---|---|---|
| SEX | Radius of gyration | Male | Female | 11.51 | 1.19e-30 | *** |
| SEX | Entropy | Male | Female | 15.62 | 5.54e-55 | *** |
| SEX | Capacity | Male | Female | 3.61 | 3.03e-04 | *** |
| AGE | Radius of gyration | age <= 29 | age between 30-54 | -4.21 | 2.59e-05 | *** |
| AGE | Radius of gyration | age <= 29 | age >=55 | 1.93 | 5.35e-02 | ns |
| AGE | Radius of gyration | age between 30-54 | age >=55 | 7.20 | 6.21e-13 | *** |
| AGE | Entropy | age <= 29 | age between 30-54 | -0.45 | 6.52e-01 | ns |
| AGE | Entropy | age <= 29 | age >=55 | -1.09 | 2.77e-01 | ns |
| AGE | Entropy | age between 30-54 | age >=55 | -0.97 | 3.33e-01 | ns |
| AGE | Capacity | age <= 29 | age between 30-54 | -2.72 | 6.44e-03 | ** |
| AGE | Capacity | age <= 29 | age >=55 | 0.53 | 5.97e-01 | ns |
| AGE | Capacity | age between 30-54 | age >=55 | 3.48 | 5.11e-04 | *** |
| INCOME | Radius of gyration | earnings <= $1250/month | earnings between $1251-3333/month | 3.20 | 1.36e-03 | ** |
| INCOME | Radius of gyration | earnings <= $1250/month | earnings >= $3333/month | -1.15 | 2.48e-01 | ns |
| INCOME | Radius of gyration | earnings between $1251-3333/month | earnings >= $3333/month | -5.77 | 7.82e-09 | *** |
| INCOME | Entropy | earnings <= $1250/month | earnings between $1251-3333/month | 0.96 | 3.37e-01 | ns |
| INCOME | Entropy | earnings <= $1250/month | earnings >= $3333/month | 7.29 | 3.24e-13 | *** |
| INCOME | Entropy | earnings between $1251-3333/month | earnings >= $3333/month | 7.73 | 1.12e-14 | *** |
| INCOME | Capacity | earnings <= $1250/month | earnings between $1251-3333/month | -10.10 | 5.73e-24 | *** |
| INCOME | Capacity | earnings <= $1250/month | earnings >= $3333/month | -10.47 | 1.16e-25 | *** |
| INCOME | Capacity | earnings between $1251-3333/month | earnings >= $3333/month | 0.71 | 4.77e-01 | ns |
| RACE | Radius of gyration | White | Black or African American | 1.31 | 1.90e-01 | ns |
| RACE | Radius of gyration | White | Asian | 5.17 | 2.55e-07 | *** |
| RACE | Radius of gyration | Black or African American | Asian | 2.56 | 1.04e-02 | * |
| RACE | Entropy | White | Black or African American | 3.45 | 5.64e-04 | *** |
| RACE | Entropy | White | Asian | 12.03 | 2.58e-32 | *** |
| RACE | Entropy | Black or African American | Asian | 6.80 | 1.18e-11 | *** |
| RACE | Capacity | White | Black or African American | 8.77 | 2.37e-18 | *** |
| RACE | Capacity | White | Asian | 18.33 | 3.47e-71 | *** |
| RACE | Capacity | Black or African American | Asian | 7.96 | 1.92e-15 | *** |
| EDUCATION | Radius of gyration | Less than high school | High school or equivalent, no college | -1.48 | 1.40e-01 | ns |
| EDUCATION | Radius of gyration | Less than high school | Some college or Associate degree | -0.63 | 5.30e-01 | ns |
| EDUCATION | Radius of gyration | Less than high school | Bachelor degree or advanced degree | 0.42 | 6.74e-01 | ns |
| EDUCATION | Radius of gyration | High school or equivalent, no college | Some college or Associate degree | 1.52 | 1.29e-01 | ns |
| EDUCATION | Radius of gyration | High school or equivalent, no college | Bachelor degree or advanced degree | 3.06 | 2.20e-03 | ** |
| EDUCATION | Radius of gyration | Some college or Associate degree | Bachelor degree or advanced degree | 1.76 | 7.77e-02 | ns |
| EDUCATION | Entropy | Less than high school | High school or equivalent, no college | -1.00 | 3.16e-01 | ns |
| EDUCATION | Entropy | Less than high school | Some college or Associate degree | 2.24 | 2.51e-02 | * |
| EDUCATION | Entropy | Less than high school | Bachelor degree or advanced degree | 10.28 | 1.01e-24 | *** |
| EDUCATION | Entropy | High school or equivalent, no college | Some college or Associate degree | 5.81 | 6.22e-09 | *** |
| EDUCATION | Entropy | High school or equivalent, no college | Bachelor degree or advanced degree | 19.30 | 6.78e-83 | *** |
| EDUCATION | Entropy | Some college or Associate degree | Bachelor degree or advanced degree | 14.93 | 2.35e-50 | *** |
| EDUCATION | Capacity | Less than high school | High school or equivalent, no college | 0.52 | 6.03e-01 | ns |
| EDUCATION | Capacity | Less than high school | Some college or Associate degree | 0.08 | 9.33e-01 | ns |
| EDUCATION | Capacity | Less than high school | Bachelor degree or advanced degree | 4.53 | 5.78e-06 | *** |
| EDUCATION | Capacity | High school or equivalent, no college | Some college or Associate degree | -0.79 | 4.30e-01 | ns |
| EDUCATION | Capacity | High school or equivalent, no college | Bachelor degree or advanced degree | 6.77 | 1.33e-11 | *** |
| EDUCATION | Capacity | Some college or Associate degree | Bachelor degree or advanced degree | 7.88 | 3.31e-15 | *** |

Table S4: Comparison to test whether there are differences between groups inside the same socio-demographic factor (e.g., male unemployed vs female unemployed). The statistical significance is computed with the Welch T-test statistic. Statistical significance is reported using the following notation for different p-values: * ($p < 0.05$), ** ($p < 0.01$), *** ($p < 0.001$), *ns* ($p >= 0.05$).

| Demographic | Mobility Metric | Group | Group avg | T-test | p-value | Significance |
|---|---|---|---|---|---|---|
| SEX | Radius of gyration | Male | -0.08 | -4.85e+01 | 0.00e+00 | *** |
| SEX | Radius of gyration | Female | -0.1 | -5.89e+01 | 0.00e+00 | *** |
| SEX | Entropy | Male | -0.56 | -2.62e+02 | 0.00e+00 | *** |
| SEX | Entropy | Female | -0.59 | -2.72e+02 | 0.00e+00 | *** |
| SEX | Capacity | Male | -0.31 | -1.69e+02 | 0.00e+00 | *** |
| SEX | Capacity | Female | -0.3 | -1.52e+02 | 0.00e+00 | *** |
| AGE | Radius of gyration | age <= 29 | -0.11 | -3.07e+01 | 1.83e-204 | *** |
| AGE | Radius of gyration | age between 30-54 | -0.09 | -6.10e+01 | 0.00e+00 | *** |
| AGE | Radius of gyration | age >=55 | -0.08 | -1.93e+01 | 5.42e-83 | *** |
| AGE | Entropy | age <= 29 | -0.62 | -1.48e+02 | 0.00e+00 | *** |
| AGE | Entropy | age between 30-54 | -0.57 | -3.19e+02 | 0.00e+00 | *** |
| AGE | Entropy | age >=55 | -0.55 | -1.33e+02 | 0.00e+00 | *** |
| AGE | Capacity | age <= 29 | -0.28 | -7.50e+01 | 0.00e+00 | *** |
| AGE | Capacity | age between 30-54 | -0.31 | -1.94e+02 | 0.00e+00 | *** |
| AGE | Capacity | age >=55 | -0.32 | -8.69e+01 | 0.00e+00 | *** |
| INCOME | Radius of gyration | earnings <= $1250/month | -0.09 | -3.14e+01 | 1.64e-214 | *** |
| INCOME | Radius of gyration | earnings between $1251-3333/month | -0.08 | -3.51e+01 | 2.36e-268 | *** |
| INCOME | Radius of gyration | earnings >= $3333/month | -0.09 | -5.64e+01 | 0.00e+00 | *** |
| INCOME | Entropy | earnings <= $1250/month | -0.57 | -1.50e+02 | 0.00e+00 | *** |
| INCOME | Entropy | earnings between $1251-3333/month | -0.57 | -2.03e+02 | 0.00e+00 | *** |
| INCOME | Entropy | earnings >= $3333/month | -0.58 | -2.78e+02 | 0.00e+00 | *** |
| INCOME | Capacity | earnings <= $1250/month | -0.32 | -9.43e+01 | 0.00e+00 | *** |
| INCOME | Capacity | earnings between $1251-3333/month | -0.27 | -1.07e+02 | 0.00e+00 | *** |
| INCOME | Capacity | earnings >= $3333/month | -0.32 | -1.76e+02 | 0.00e+00 | *** |
| RACE | Radius of gyration | White | -0.09 | -7.55e+01 | 0.00e+00 | *** |
| RACE | Radius of gyration | Black or African American | 0.06 | 1.55e+00 | 1.22e-01 | ns |
| RACE | Radius of gyration | Asian | 0.5 | 1.73e+00 | 8.43e-02 | ns |
| RACE | Entropy | White | -0.57 | -3.71e+02 | 0.00e+00 | *** |
| RACE | Entropy | Black or African American | -0.6 | -4.29e+01 | 9.39e-323 | *** |
| RACE | Entropy | Asian | -0.58 | -1.94e+01 | 3.50e-77 | *** |
| RACE | Capacity | White | -0.31 | -2.25e+02 | 0.00e+00 | *** |
| RACE | Capacity | Black or African American | -0.34 | -3.36e+01 | 8.91e-226 | *** |
| RACE | Capacity | Asian | -0.32 | -2.13e+01 | 2.14e-93 | *** |
| EDUCATION | Radius of gyration | Less than high school | -0.05 | -6.82e+00 | 9.38e-12 | *** |
| EDUCATION | Radius of gyration | High school or equivalent, no college | -0.08 | -3.57e+01 | 6.58e-277 | *** |
| EDUCATION | Radius of gyration | Some college or Associate degree | -0.09 | -4.58e+01 | 0.00e+00 | *** |
| EDUCATION | Radius of gyration | Bachelor degree or advanced degree | -0.11 | -4.74e+01 | 0.00e+00 | *** |
| EDUCATION | Entropy | Less than high school | -0.62 | -7.08e+01 | 0.00e+00 | *** |
| EDUCATION | Entropy | High school or equivalent, no college | -0.56 | -1.92e+02 | 0.00e+00 | *** |
| EDUCATION | Entropy | Some college or Associate degree | -0.56 | -2.28e+02 | 0.00e+00 | *** |
| EDUCATION | Entropy | Bachelor degree or advanced degree | -0.6 | -2.12e+02 | 0.00e+00 | *** |
| EDUCATION | Capacity | Less than high school | -0.32 | -5.74e+01 | 0.00e+00 | *** |
| EDUCATION | Capacity | High school or equivalent, no college | -0.31 | -1.26e+02 | 0.00e+00 | *** |
| EDUCATION | Capacity | Some college or Associate degree | -0.31 | -1.39e+02 | 0.00e+00 | *** |
| EDUCATION | Capacity | Bachelor degree or advanced degree | -0.29 | -1.11e+02 | 0.00e+00 | *** |

Table S5: Comparison of the groups of a socio-demographic factor against their corresponding employed population (e.g., male unemployed vs male employed). The statistical significance is computed with the T-test statistic. Statistical significance is reported using the following notation for different p-values: * ($p < 0.05$), ** ($p < 0.01$), *** ($p < 0.001$), *ns* ($p >= 0.05$).

# Methods S1: Datasets

## S1.1 GPS location data

The location data is provided by Cuebiq Inc., a location intelligence and measurement company. The dataset was shared within the Cuebiq Data for Good program, which provides access to de-identified and anonymized mobility data for academic and research purposes.

The location data provided consists of privacy-enhanced GPS locations for research purposes, from January 2020 to September 2020, and includes only users who have opted-in to share their data anonymously. The data is General Data Protection Regulation (GDPR) and California Consumer Privacy Act (CCPA) compliant. Furthermore, to increase and preserve users' privacy, Cuebiq obfuscates home and work locations to the Census Block Group level. The data is collected through the Cuebiq Software Development Kit (SDK) which collects user locations through GPS and Wi-Fi signals in Android and iOS devices.

The accuracy of location data is determined by the device and varies from 0 to over 100 meters. As shown in the Fig. S1(left), the accuracy distribution of the original GPS events (before processing them with our stop location algorithm) in our dataset is bimodal, with one peak around 5 meters and another at approximately 65 meters. While the first peak highlights the high quality of the overall data employed, we speculate that the latter peak is caused by the home obfuscation mechanism implemented by the data provider to preserve user privacy [2]. Additionally, Fig. S1(right) illustrates the average number of hours per day in which at least one GPS event is recorded per user. The data indicate that most users have coverage across almost all hours of the day, allowing us to reliably capture human mobility patterns.

## S1.2 Longitudinal Employer-Household Dynamics (LEHD)

The Longitudinal Employer-Household Dynamics (LEHD) program of the US Census Bureau produces public-use information about employers and employees by combining federal, state, and Census Bureau data. Socio-economic information is used by state and local authorities to make informed decisions for their economies. We use three different surveys provided by this program in order to assign in probability industrial sectors to each individual analysed, according to the North American Industry Classification System (NAICS). NAICS represents the standard classification of businesses used by Federal Agencies in the United States for the purposes of collecting and analysing statistical data related to the US business economy. This study uses the LEHD Origin-Destination Employment Statistics (LODES) datasets, which include three different types of data: Origin-Destination (OD), Residence Area Characteristics (RAC), and Workplace Area Characteristics (WAC). The data was collected at the census block geographic level in 2018. LODES statistics contain data about geographic employment patterns by workplace and residential locations, and they include the age of the worker, earnings, industry, sex, race, ethnicity, and education. Therefore, LODES provide geographical statistics about employers and employees and can be used to answer questions about spatial, economic, and demographic issues related to workplaces and home-to-work flows. The Residence Area Characteristics (RAC) and the Workplace Area Characteristics (WAC) datasets provide statistics about the total number of jobs, the total number of jobs in each NAICS sector, and also according to age, earnings of the workers, race, ethnicity, education, and sex. The difference between the RAC and the WAC datasets is that the first computes these statistics according to the home census block, while in the WAC dataset jobs are totalled by the work census block. The Origin-Destination (OD) dataset provides information considering both home and work census blocks. It includes the home and work GEOIDs and the same statistics of the WAC and the RAC datasets. However, different from the WAC and the RAC datasets, the OD dataset provides the total number of jobs according to three macro-sectors, which are: Goods Producing industry sectors, Trade, Transportation, and Utilities industry sectors, and All Other Services industry sectors. These macro-sectors can be matched with the 20 NAICS sectors to combine the WAC, the RAC, and the OD datasets.

## S1.3 Unemployment Insurance (UI) claims

Unemployment Insurance (UI) programs are organised at the state level and they have the aim to provide assistance to jobless people who are looking for work. UI data provides the number of submitted claims

divided by industry sectors (NAICS) and allows tracking of employment changes at the state level. This study uses the dataset ETA 203 - Characteristics of the Insured Unemployed of the UI program[1]. It provides information about Unemployed Insurance claimants for each state for each month. It describes how the population of claimants varies over time and it reports characteristics about sex, race/ethnicity, age, industry, occupation, etc.

### S1.4 Local Area Unemployment Statistics (LAUS)

The Local Area Unemployment Statistics (LAUS) program of the Bureau of Labor Statistics (BLS)[2] provides monthly estimates of total employment and unemployment across different geographic levels (states, metropolitan areas, counties, etc). The employment and unemployment data are estimated by combining current and historical data from the Current Population Survey (CPS), the Current Employment Statistics (CES), and state Unemployment Insurance (UI) data. An estimation process done by the LAUS program produces the official unemployment rate.

### S1.5 Employment data from the Bureau of Labor Statistics (BLS)

The Bureau of Labor Statistics (BLS) is a unit of the Department of Labor of the United States that measures several economic aspects, such as labour market activity, working conditions, price changes, and productivity in the US economy. In this work, we incorporate state-level employment information obtained from the Bureau of Labor Statistics (BLS) through the Quarterly Census of Employment and Wages (QCEW) program. The program provides employment and wage information reported by employers with a coverage of more than 95 percent of US jobs. The data is available at the county, MSA, state, and national levels divided by NAICS sectors[3].

### S1.6 Remote Workability

To include in our methodology for inferring the unemployment status of an individual the information about the teleworkability of a job, we leverage the work done by Dingel et al. [1]. In their work, the authors developed a methodology and provided a dataset that contains the probability of working from home given the industrial sector (NAICS) an individual works in. The probabilities are reported in Tab. S1.

## Methods S2: Sample composition

Samples of users gathered as passively collected GPS trajectories from individuals' personal devices, such as mobile phones, are often afflicted by different biases making them not representative of a population. Nevertheless, it is possible, leveraging local demographic information, to partially remove and account for these biases getting closer to what could be a fair representation of a country's population.

### S2.1 Post-stratification reweighting: controlling biases in mobile phone data

As described in the manuscript, residential and workplace detection is of paramount importance in the inference of workers' industrial sectors. However, there is more that can be done to better exploit the joint information of GPS trajectories and demographic information available from census data. In particular, when analyzing GPS location data, it has become more and more clear the importance of de-biasing personal device data from uneven user base distributions [4, 3]. To this end, we leverage demographic information at the census block group level to reconstruct a population-representative base of individuals. This is performed by an individual-based reweighting step which leverages the detection of the individual residential areas (e.g., see [4, 3]). Representativity is ensured by using state-specific stratified population information and by reweighting upsampled individuals from census block groups where at least one individual in our data is residing based on the specific strata population. Single individual weights are assigned based on the

[1] https://oui.doleta.gov/unemploy/DataDownloads.asp
[2] https://www.bls.gov/lau/
[3] https://www.bls.gov/cew/

fraction of the stratified population of each census block group as well as on the state-specific workforce distribution across the different CNS.

To compute sample weights, we applied a two-dimensional proportional weighting approach. This weighting method aligns the mobile-phone user-base sample distribution with the known census population distributions at two distinct levels. The geographic level: within each census block group (identified by GEOIDs), we compute the geographic weight as the ratio between the true population proportion (from census data) and the proportion of mobile-phone users observed residing within that unit:

$$w_{GEOID} = \frac{\text{Census Population}_{GEOID}/\text{Total Census Population}}{\text{Users}_{GEOID}/\text{Total Users}}$$

Occupational (CNS) level: at the state level, we additionally adjust for occupational sectors based on the proportion of the workforce in each occupational category (CNS sectors). The occupational weight is thus calculated as:

$$w_{CNS,state} = \frac{\text{Census Workforce}_{CNS,state}/\text{Total Census Workforce}_{state}}{\text{Users}_{CNS,state}/\text{Total Users}_{state}}$$

The final weight for each individual mobile-phone user is the product of these two proportional weights, thus simultaneously correcting for geographic and occupational biases:

$$w_{final} = w_{geo} \times w_{occ}$$

Figure S2 and Figure S3 report the weights' distributions.

Once the weight are computed, the following procedure is employed to enrich individual data: i) single individuals are upsampled to enrich our dataset; ii) upsampled individuals are assigned, based on GEOID-specific demographic and industry sector data, information about their probable sex, age group, income level, race, education level, and industrial sector; iii) individual weights are independently assigned for each of the demographic and work sector information based on the strata distribution at the state level; iv) weights are used for bootstrapping to construct a synthetic representative population consisting of $500,000$ individuals for each state included in the study. For population-wide analyses, each individual is treated in our analysis as an independent data point. In this situation, stop location sequences are aggregated together, providing a representative picture of employed/unemployed behavioural differences. In contrast, strata-specific results report group-specific behaviour whose representativity is controlled for by computing errors exclusively including originally independent stop-location sequences.

Additionally, to extend the validity of our results we aggregate results from multiple states displaced across different geographical areas of the US selecting them based on the internal repartition of their workforce into the three main economic sectors (see SI S2.2).

## S2.2 States selection

To improve the data representation further, we focus our analysis on seven US states: New York, Wyoming, Indiana, Idaho, Washington, North Dakota, and New Mexico. These states have been selected to take into account at the same time:

- the representation of different workforce compositions in terms of population distribution across primary, secondary and tertiary economic sectors. Fig. S4 shows the distribution of the states taking into account the percentages of the primary, secondary and tertiary workforce in each US states. We selected the states in order to cover the entire space. The information about the economics sector are derived from the NAICS sector data and mapped according to Tab. S2;
- the geographical representation of the states to avoid the selection of states the belongs to a single geographic region (e.g., Northeast, etc.). Fig. S5 depicts the selected states' geographical distribution.

# Methods S3: Job loss detection

## S3.1 Remote work adjustment

In this section, we explain in greater detail the procedure to assign to each sector at each time $t$ a probability of working remotely (or being unemployed). Following the idea described in the main text, we aim to properly define a probability that accounts for both the amount of work that can still be performed from remote, $\tilde{R}_s(t)$, and the reduction work that was performed in person during the baseline period (which is either now performed by employees working remotely or not performed anymore, thus making those individual unemployed), $\tilde{r}_s(t)$. Both these quantities express sector-specific aggregate measures to avoid further assumptions, needed, for example, in case we would want to build an individual probability measure.

The probability presented in the main text ($P_s(t)$, we report the definition below) uses the ratio between the remaining work time that could be performed from remote and the reduction in in-presence work time as a conservative measure, likely to undershoot the probability of being unemployed.

$$P_s(t) = 1 - \min\left(\frac{\tilde{R}_s(t)}{\tilde{r}_s(t)}, 1\right)$$

The measure is bounded between 0, in the case where $\tilde{r}_s(t) \leq \tilde{R}_s(t)$ (i.e. when the amount of time that can still be worked from remote exceeds the reduction of in-person work time), and 1, in the case where $\tilde{R}_s(t) = 0$ (i.e. when all the remote-work time is already accounted for by "not at risk" individuals, those with $rt_s(t) < 1$). The aim of this section is to introduce the formal definition of both quantities ($\tilde{R}_s(t)$ and $\tilde{r}_s(t)$) and to provide a more clear understanding of how those quantities are computed. To this end, we report here the definitions introduced in the main text of:

$$\begin{cases} r_u(t) = 1 & \text{if } v_u(t) = 0 \\ r_u(t) = 0 & \text{otherwise} \end{cases}$$

(being $v_u(t)$ the number of visits $u$ made to their work location within the time window $t$), and

$$rt_u(t) = 1 - \frac{w_u(t)}{w_u(b)}.$$

with $w_u(t)$ being the time the user $u$ spent at the workplace in the time window $t$, and $w_u(b)$ being the median of the time spent at work (within windows of the same size as $t$) during the baseline period (Jan 3 - Mar 7, 2020).

### S3.1.1 Adjusted sector remote work fraction: $\tilde{R}_s(t)$

$\tilde{R}_s(t)$ is defined for each time window $t$ by the following formula:

$$\tilde{R}_s(t) = \max\left(0, R_s - W_s(t)\right);$$

where $R_s$ is the fraction of work that can be performed remotely, and $W_s(t)$ is the weighted fraction of work performed at home by individuals who are still commuting at time $t$. Formally, $R_s$ is a quantity taken from the literature for each different NAICS (see Dingel et al. [1]), while $W_s(t) = \sum_{u' \in s} rt'_u(t) * rw'_{u_i}$. Here $rw_u$ is an individual-specific weight. It is proportional to the fraction of time the individual was spending at work with respect to the amount of time spent at work by the entire sector workforce during the baseline period. Formally it is defined as:

$$rw_u = \frac{\bar{w}_u(b)}{\sum_{u' \in s} \bar{w}_{u'}(b)}$$

where $\bar{w}_u(b)$ is the average time spent at work in a day by a single individual during the baseline period $b$.

### S3.1.2 Weighted reduction in in-person work time: $\tilde{r}_s(t)$

Combining $r_u(t)$ and $rt_u(t)$ with $rw_u$, as defined above we can formalize the concept of "weighted fraction of remaining work time that could be performed remotely by those individuals who stopped visiting their work location". The decision whether this time will correspond effectively to remote work time is encoded in the formula for the unemployment probability, here we focus on formalizing the $\tilde{r}_s(t)$ mathematical definition:

$$\tilde{r}_s(t) = \sum_{u' \in s} rt_{u'}(t) * rw_{u'} - \sum_{u' \in s} (1 - r_{u'}(t)) * rt_{u'}(t) * rw_{u_i}.$$

The first term captures the sector-specific weighted reduction in the amount of time spent at work by the entire workforce population within each time window $t$. The second term represents the weighted reduction of time spent at work by those individuals who are still going to their workplace in $t$. Thus, the difference between the two represents the total weighted reduction in time spent at work in $t$ for those individuals. A complementary definition would be to directly refer to the weighted reduction of "at-risk" individuals: $\tilde{r}_s(t) = \sum_{u' \in s} r_{u'}(t) * rw_{u_i}$. The efficacy of this procedure is to easily account for systemic reductions in the in-presence work time alongside the intrinsic industrial sector remote workability.

### S3.1.3 Remote workability mechanism: an intuition

The idea behind this procedure is to use $rt_u(t) * rw_u$ to re-weight the risk of unemployment for each user $u$ in each time window $t$ to take into account the fraction of visits/work time that particular user was "consuming" during the baseline compared to all other users in a specific window and within the same NAICS. Intuitively, by aggregating $rt_u(t) * rw_u$ at the sector level for all users that are still visiting their work location ($r_u(t) < 1$), we are computing a weighted average (over the fraction of time each user worked) of the reduction of the time spent at work ($rt_u(t)$). This can also be seen as the change in remote work that we measure from a reduction in the time spent at work by users that are still visiting their work location, i.e. a measure of how much remote work is already performed by individuals who are still employed. Figure S6 and Figure S7 show how this procedure act on the different industrial sectors based on their remote workability levels (less teleworkable and more teleworkable, respectively).

# Methods S4: Algorithm evaluation

In this section, we detail the performance of our unemployment detection algorithm. Given the differences in data sources between Unemployment Insurance (UI) claim counts and the official unemployment figures from the Local Area Unemployment Statistics (LAUS) program (see Section ), we evaluate the algorithm using both datasets. We chose these datasets for their differing temporal and spatial resolutions:

- *Temporal Resolution*: UI claims data are reported weekly, providing a more immediate, near real-time basis for evaluating algorithm performance. By contrast, LAUS data represent official unemployment figures calculated through an estimation process, incorporating multiple sources (including UI claims) and are published with a lag.

- *Geographic Resolution*: UI claims are available at the state level, segmented by NAICS sectors, which allows sector-specific performance analysis. Meanwhile, LAUS data support a finer geographic resolution, enabling us to evaluate our algorithm at the county level.

To assess accuracy, we calculate the Pearson correlation coefficient between the monthly unemployment rates estimated by our algorithm and the corresponding unemployment rates from the UI claims at the state level ($\rho = 0.88$, see Fig. S8). We further provide sector-specific correlations for NAICS sectors (see Fig. S9). Using the LAUS data, we calculate the Pearson correlation between our algorithm's monthly unemployment rate estimates and the official LAUS unemployment rates at the state level ($\rho = 0.72$, see Fig. S10). Additionally, we present finer-grained correlations at the county level (see Fig. S11) and provide monthly-level correlations (see Fig. S12).

## Methods S5: Demographic differences

In this section we disaggregate the mobility behaviour of unemployed individuals ($t > 0$) based on the individual's socio-demographic group. Socio-demographic information is extracted from the Longitudinal Employer-Household Dynamics (LODES) dataset and includes Sex, Age, Income, Race, and Education. We provide, for each mobility metric, namely radius of gyration (SI S13- S17), entropy (SI S18- S22) and capacity (SI S23- S27), the z-scores of the metric over time and their distributions divided by a specific socio-demographic indicator. Moreover, for each mobility metric, we provide statistical tests for (i) the comparison of the groups of a socio-demographic against the entire employed population (SI Tab. S3); (ii) the comparison of the groups of a socio-demographic against their corresponding employed population (e.g., male unemployed vs male employed) (SI Tab. S5); and (iii) the comparison to test whether there are differences between groups inside the same socio-demographic factor (e.g., male unemployed vs female unemployed) (SI Tab. S4).

# Methods S6: Group specific behaviour: robustness analysis

In this section, we report three different robustness checks intended to provide additional evidence of a systematic disparity in individual mobility behaviour at the turn of the time of job loss. These analyses follow the same rationale as the analysis reported in the main manuscript (Figure 3 in particular).

## S6.1 The impact of job loss in early and late pandemic periods

We stratify the user base into two different groups: one consisting of individuals who experienced a job-loss event during the early pandemic period (from March 11, 2020, until May 22, 2020) and during the latest pandemic period covered by the mobility data (May 23, 2020 until August 2, 2020). In Figure S28 , we show group-specific mobility metrics as a function of the number of days since the date of unemployment. Consistent significant reduction in long-term mobility behaviour is obtained both for early and late pandemic. The early pandemic period shows an earlier reduction signal, while the late pandemic period shows a jump in the radius and entropy metric of a size comparable with the window size. Speculatively, this might be caused by seasonal effects of short-term unemployment systematic misclassification, which rapidly disappear as soon as the time since unemployment increases.

## S6.2 The impact of job loss in high/low baseline work activity

We stratify the user base into two different groups: one consisting of individuals who have higher-frequency work location visit patterns and the other with lower-frequency work location visit patterns. Higher/lower frequency of work location visits is defined based on the median number of days individuals visited their work location in any of the baseline windows. In Figure S29 , we show group-specific mobility metrics as a function of the number of days since the date of unemployment. Consistent significant reductions in mobility behaviour are found both for high and low-frequency work visit patterns. Low-frequency visits to the workplace show, on average, only minor differences with high-frequency individuals. While this difference is visible both in the radius of gyration and in the time allocation entropy, it also suggests that potential misclassifications due to confounding absence from work with unemployment are limited.

## S6.3 The impact of job loss in high/low teleworkable sectors

We stratify the user base into two different groups: one consisting of individuals who are inferred working on a CNS with high teleworkability, the other with lower teleworkability. The two groups are constructed leveraging the teleworkability limits by sectors as reported in Dingel et al [1]. High teleworkability groups are defined by grouping together all individuals from the seven most teleworkable CNS. Similarly, the low teleworkability group is defined by grouping together the seven less teleworkable CNS. Specifically, based on Tab. S1, we consider low-teleworkable CNS the following: CNS01, CNS04, CNS05, CNS07, CNS08, CNS16, CNS18. Similarly, we consider as high-teleworkable CNS the following: CNS06, CNS09, CNS10, CNS11, CNS12, CNS13, CNS15. . The mobility behaviour of these two groups is then compared in Fig. S30. The Figure shows group-specific mobility metrics as a function of the number of days since the date of unemployment. Consistent significant reductions in mobility behaviour are found both for high and low teleworkability groups.

## References


[1] Jonathan I Dingel and Brent Neiman. How many jobs can be done at home? *Journal of Public Economics*, 189:104235, 2020.

[2] Lorenzo Lucchini, Simone Centellegher, Luca Pappalardo, Riccardo Gallotti, Filippo Privitera, Bruno Lepri, and Marco De Nadai. Living in a pandemic: changes in mobility routines, social activity and adherence to covid-19 protective measures. *Scientific reports*, 11(1):24452, 2021.

[3] Lorenzo Lucchini, Ollin D. Langle-Chimal, Lorenzo Candeago, Lucio Melito, Alex Chunet, Aleister Montfort, Bruno Lepri, Nancy Lozano-Gracia, and Samuel P. Fraiberger. Socioeconomic disparities in mobility behavior during the covid-19 pandemic in developing countries. *EPJ Data Science*, 14(1):25, Mar 2025.

[4] Takahiro Yabe, Bernardo García Bulle Bueno, Xiaowen Dong, Alex Pentland, and Esteban Moro. Behavioral changes during the covid-19 pandemic decreased income diversity of urban encounters. *Nature Communications*, 14(1):2310, 2023.
